# Perception by Palpation: Development and Testing of a Haptic Ferrogranular Jamming Surface

**DOI:** 10.3389/frobt.2021.745234

**Published:** 2021-09-28

**Authors:** Sigurd Bjarne Rørvik, Marius Auflem, Henrikke Dybvik, Martin Steinert

**Affiliations:** TrollLABS, Department of Mechanical and Industrial Engineering, Faculty of Engineering, Norwegian University of Science and Technology (NTNU), Trondheim, Norway

**Keywords:** haptic interface, tactile surface, simulation, palpation, granular jamming, tactile perception, ferromagnetic granules

## Abstract

Tactile hands-only training is particularly important for medical palpation. Generally, equipment for palpation training is expensive, static, or provides too few study cases to practice on. We have therefore developed a novel haptic surface concept for palpation training, using ferrogranular jamming. The concept’s design consists of a tactile field spanning 260 x 160 mm, and uses ferromagnetic granules to alter shape, position, and hardness of palpable irregularities. Granules are enclosed in a compliant vacuum-sealed chamber connected to a pneumatic system. A variety of geometric shapes (output) can be obtained by manipulating and arranging granules with permanent magnets. The tactile hardness of the palpable output can be controlled by adjusting the chamber’s vacuum level. A psychophysical experiment (N = 28) investigated how people interact with the palpable surface and evaluated the proposed concept. Untrained participants characterized irregularities with different position, form, and hardness through palpation, and their performance was evaluated. A baseline (no irregularity) was compared to three irregularity conditions: two circular shapes with different hardness (Hard Lump and Soft Lump), and an Annulus shape. 100% of participants correctly identified an irregularity in the three irregularity conditions, whereas 78.6% correctly identified baseline. Overall agreement between participants was high (κ= 0.723). The Intersection over Union (IoU) for participants sketched outline over the actual shape was IoU *Mdn* = 79.3% for Soft Lump, IoU *Mdn* = 68.8% for Annulus, and IoU *Mdn* = 76.7% for Hard Lump. The distance from actual to drawn center was *Mdn* = 6.4 mm (Soft Lump), *Mdn* = 5.3 mm (Annulus), and Mdn = 7.4 mm (Hard Lump), which are small distances compared to the size of the field. The participants subjectively evaluated Soft Lump to be significantly softer than Hard Lump and Annulus. Moreover, 71% of participants thought they improved their palpation skills throughout the experiment. Together, these results show that the concept can render irregularities with different position, form, and hardness, and that users are able to locate and characterize these through palpation. Participants experienced an improvement in palpation skills throughout the experiment, which indicates the concepts feasibility as a palpation training device.

## 1 Introduction

In simulated training environments (i.e., augmented, virtual, and mixed reality), realistic rendering of tactile interactions with the physical world is challenging, yet meaningful. This is because haptic interfaces enabling such tactile interactions must complement (and reflect) the vivid audiovisual feedback provided by the simulation (Woodrum et al., 2006). This combination could yield deeper immersion and thus facilitate the transfer of tactile experiences when transitioning to real-world scenarios. Furthermore, by realistically bridging the physical and digital world, users can develop, improve, and maintain critical psychomotor skills (Lathan et al., 2002; Zhou et al., 2012; Zhao et al., 2020). Hence, haptic interfaces in simulation can enable safe, repetitive, and available training alternatives for various professions that require dexterous hands-on experience (Carruth, 2017; Lelevé et al., 2020).

In a medical context, simulation can help narrow the gap of required clinical experience and mitigate the risk of harming or providing unsatisfactory patient treatment. However, various medical procedures require not only hands-on, but hands-only training. One of these procedures is palpation, which is used to examine a patient through touch. By palpation, diagnosis is based on tactile findings such as irregularities (lumps, fluids, tenderness) and locating pain-points. Unfortunately, common equipment such as wearable tactile devices and kinesthetic devices are less suited in this use-case given their current resolution, Degrees of Freedom (DOF), and tactile limitations (Licona et al., 2020). Consequently, simulated palpation exercises are mainly performed using static case-specific models (phantoms) or mannequins (patient simulators). While these can provide safe and repetitive training conditions, their fixed number of study cases, task-specific functionalities, and limited tactile realism are collectively obstacles for current healthcare training and education.

Haptic interfaces designed for palpation training should enable users to practice locating and describing tactile irregularities, as they would when palpating a real patient. Hence, multiple tactile displays are promising in this context by utilizing technology ranging from pin arrays (Wagner et al., 2002), to shape memory alloy actuators (Taylor et al., 1998) and airborne ultrasound (Iwamoto et al., 2008). However, such solutions are generally expensive, complex in operation and non-continuously available, thus limiting their use and widespread in research and education. Moreover, as these solutions rely on using a matrix of actuators or tactile outputs, it restricts the obtainable resolution, scalability and robustness of such interfaces. Furthermore, compliance and flexibility are often compromised by using rigid mechanisms to achieve haptic feedback. Therefore, attention has been brought to using soft robotics principles for haptic applications, as these can approximate soft body animations and organic behaviors suitable to medical training, among others (Manti et al., 2016).

**FIGURE 1:**
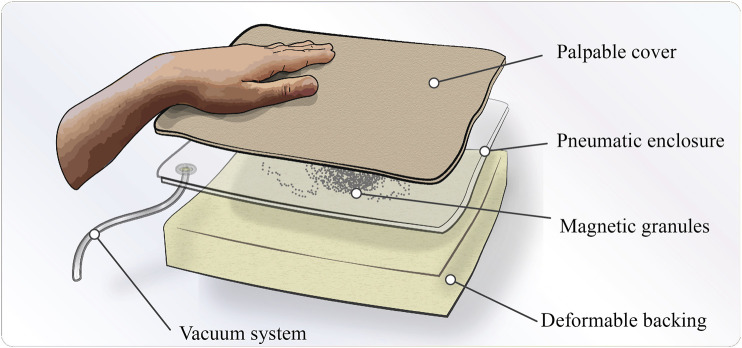
Descriptive illustration of the haptic interface concept.

An interesting area of soft robotics for medical training applications is the use of granular jamming mechanisms for haptic feedback. Granular jamming enables interfaces to alter stiffness and thus simulate compliant objects with variable hardness. This technology has been explored in medical training devices as embedded tactile modules (He et al., 2021), multi-fingered palpation interfaces (Li et al., 2014), and as actuation to enable objects and surfaces to alter shape and hardness for palpation (Stanley et al., 2016; Koehler et al., 2020). While this technology looks promising, current solutions often require complex pneumatic systems, since a matrix of actuated cells or objects is needed. Thus, this could limit the tactile resolution and geometrical freedom of rendered objects. Based on this existing work on granular jamming interfaces, we have developed a simple and low-cost technology utilizing ferromagnetic granulate. Our technology enables the granules to be remotely manipulated in an unjammed state and thus create customized tactile objects. Furthermore, when jammed, the hardness of these objects can be altered by the applied vacuum, i.e., how firmly the granules are packed together in a sealed chamber. In a haptic interface prototype described in Figure 1, the ferrogranular jamming principle is used to render palpable irregularities between two compliant layers. The prototype was developed to examine the feasibility and usability of this technology in a tactile display application. Moreover, this technology could be used to challenge the complexity, accessibility and cost of current haptic interfaces.

This work relates to the existing literature on tactile interactions, and more precisely, users’ tactile perception of hardness and geometrical shapes. Hence, studies investigating the psychophysical perception of hardness and shapes have been of interest (Tan et al., 1992; Srinivasan and LaMotte, 1995; Bergmann Tiest and Kappers, 2009; Frisoli et al., 2011). However, the use-case of palpable interfaces that requires a perceptual exploration and manipulation is a less explored area with fewer examples (Lederman and Klatzky, 1993; Genecov et al., 2014). As this encourages more research on users’ interaction and performance using haptic interfaces, our conceptual prototype has been piloted in a palpation experiment. This experiment investigates whether untrained users can locate and determine the form and hardness of rendered irregularities by palpation. Information of hardness, speed (time used to find irregularity) and accuracy of form and position has been collected, together with users’ subjective experience throughout the experiment.

This paper examines using soft-robotics principles to alter the characteristics of a haptic interface for medical diagnostics training. This investigation has resulted in the concept shown in Figure 1, which uses granular jamming and ferromagnetic granulate manipulation to achieve various palpable outputs. The concept is used to assess untrained users’ ability to locate and characterize the shape and hardness of different irregularities using palpation. Considering this concept for a novel haptic interface and the context of medical palpation training, we try to answer the following research questions in this paper:

i. Can the novel ferrogranular jamming concept be used as a haptic interface for palpation exercises?
ii. How well can untrained users determine the position, form and hardness of irregularities rendered by the haptic interface using palpation techniques?
iii. Did participants think their palpation skills improved during the experiment?

## 2 Materials: Design of the Ferrogranular Jamming Interface

This chapter starts with a short introduction to the ferrogranular jammer. Secondly, the theory of granular jamming and magnetic manipulation is presented. Lastly, the manufacturing of the magnetic granules and chamber is presented before the pneumatic setup.

**FIGURE 2:**
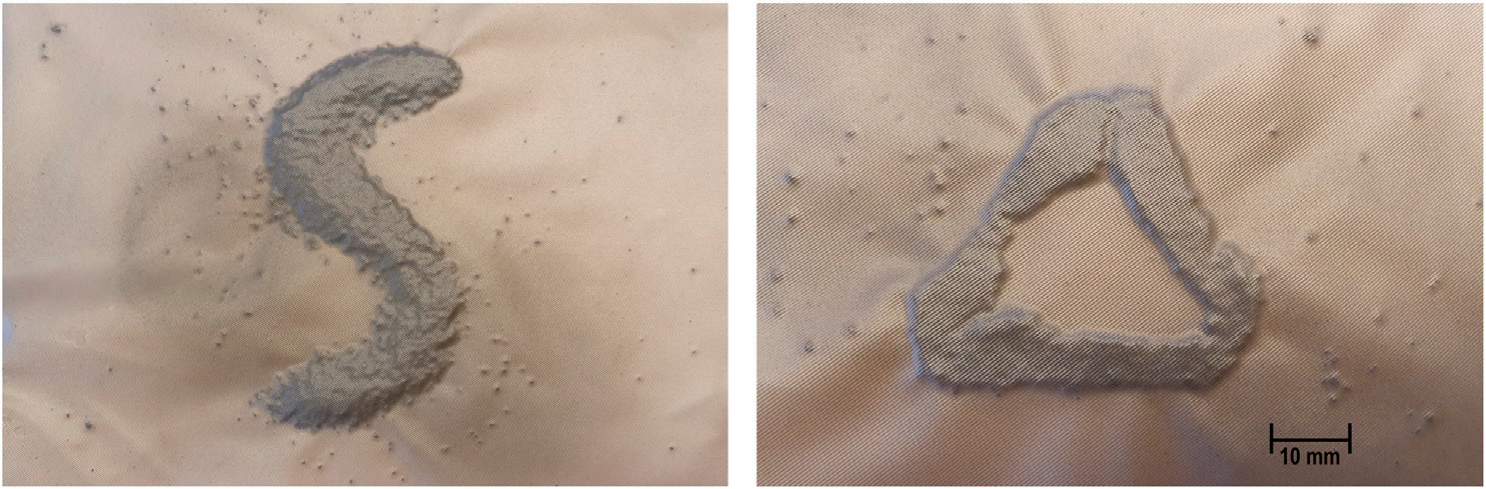
Pictures of two arrangement possibilities.

The prototype was developed to examine the feasibility and usability of a ferrogranular jamming interface in a tactile display for palpation. The novelty of the proposed concept is the introduction of magnetic manipulation of granules in a jamming application. This innovation provides the opportunity to manipulate the granular media inside a compliant vacuum chamber, thus managing the position, form and hardness of the palpable outputs. Some examples are shown in Figure 2, where the jammed granulate shapes are visible within the translucent chamber. To act as a deformable and palpable structure the vacuum chamber is sandwiched between a deformable polyurethane (PU) foam backing (60 mm) and a flexible polyethylene (PE) fabric cover (4 mm) (as seen in Figure 5B).

### 2.1 Granular Jamming and Magnetic Manipulation

Granular jamming works by transitioning granular matter from a low-density compliant packing to a high-density rigid packing. This change is done by removing the fluid/medium surrounding the granulate, which produces an external hydrostatic pressure. From this, the granules can behave both like a fluid and a solid. When the granules are in a low-density packing, the intergranular friction is low, resulting in a fluid-like state. Vice versa, when the vacuum level increases, higher intergranular friction results in a jammed and solid-like state. In the jammed state, the granules distributes applied force through the grains so that the group of particles functions as a stiff and compliant material (Cates et al., 1998).

Particle jamming has been a big research topic for engineers and material scientists for the last few decades. The principle of reversibly transitioning the granular media from a fluid-like state to a more rigid state has been seen to be applicable to various domains, such as industrial grippers (Harada et al., 2016; D’Avella et al., 2020), minimally invasive surgery (Jiang et al., 2012) and robotic locomotion (Steltz et al., 2009). Granular jamming is a prevalent type of actuation within soft robotics applications because of two main reasons: 1) considerable stiffness variation with little volume change, and 2) possibility to adjust the stiffness variability area so it can be easily adapted to different soft robotics applications (Fitzgerald et al., 2020).

There has been research on optimizing granules for granular jamming with different aspects; size, shape and volume fraction (Jiang et al., 2012), chamber material (Jiang et al., 2012) and using soft granules (Putzu, Konstantinova, and Althoefer 2019). However, a common feature for these studies is the stasis of the granulate. To the best of our knowledge, there has been no research focusing on the movability of granules in a jamming context. For example, Follmer et al. (2012) reviewed jamming in a user-interface context, where none of the technologies utilized movement of the granules.

Using magnetic fields is an effective way to transport and position magnetic particles in a medium. The most prominent concept of ferromagnetic particles in a fluid is ferrofluid. This colloidal liquid consists of surfactant-coated magnetic particles with a size order of 10 nm suspended in a liquid medium. When the fluid is subjected to a magnetic field, it forms a shape like the magnetic field and acts more like a solid. Generally, ferromagnetic particles are induced by two types of interaction energy: the one between the particles and the magnetic field E^H^, and between particles E^M^ (Cao et al., 2014). Using a magnetic field to manipulate magnetic particles has been used in microfluidic systems, such as magnetorheological fluid in user interfaces (Hook et al., 2009; Jansen et al., 2010) and biological analysis and catalysis (Gijs et al., 2010).

The advantages of using ferromagnetic granules include: 1) Controllability—Ferro-granulate can be arranged numerous ways by designing magnetic fields. 2) Noncontact—Magnetic particles can be remotely manipulated. 3) Precision—Ferromagnetic granules can be placed at a target region with high precision by precisely designing a magnetic field with local maximum field strength at preferred areas (Cao et al., 2014).

### 2.2 Manufacturing of Magnetic Granulate

Based on the previous research done on granular jamming, manufacturing of ferromagnetic granules to be used in a haptic interface were investigated. A central factor for the granulate in this research is how high interparticle friction yields higher viscosity in the un-jammed state but yields higher hardness when jammed and vice versa. Since moving the granules in the unjammed state is essential, we investigated the granule material and manufacturing methods that produce granules with lower interparticle friction in the unjammed state but still yielding sufficient hardness in the jammed state.

**FIGURE 3:**
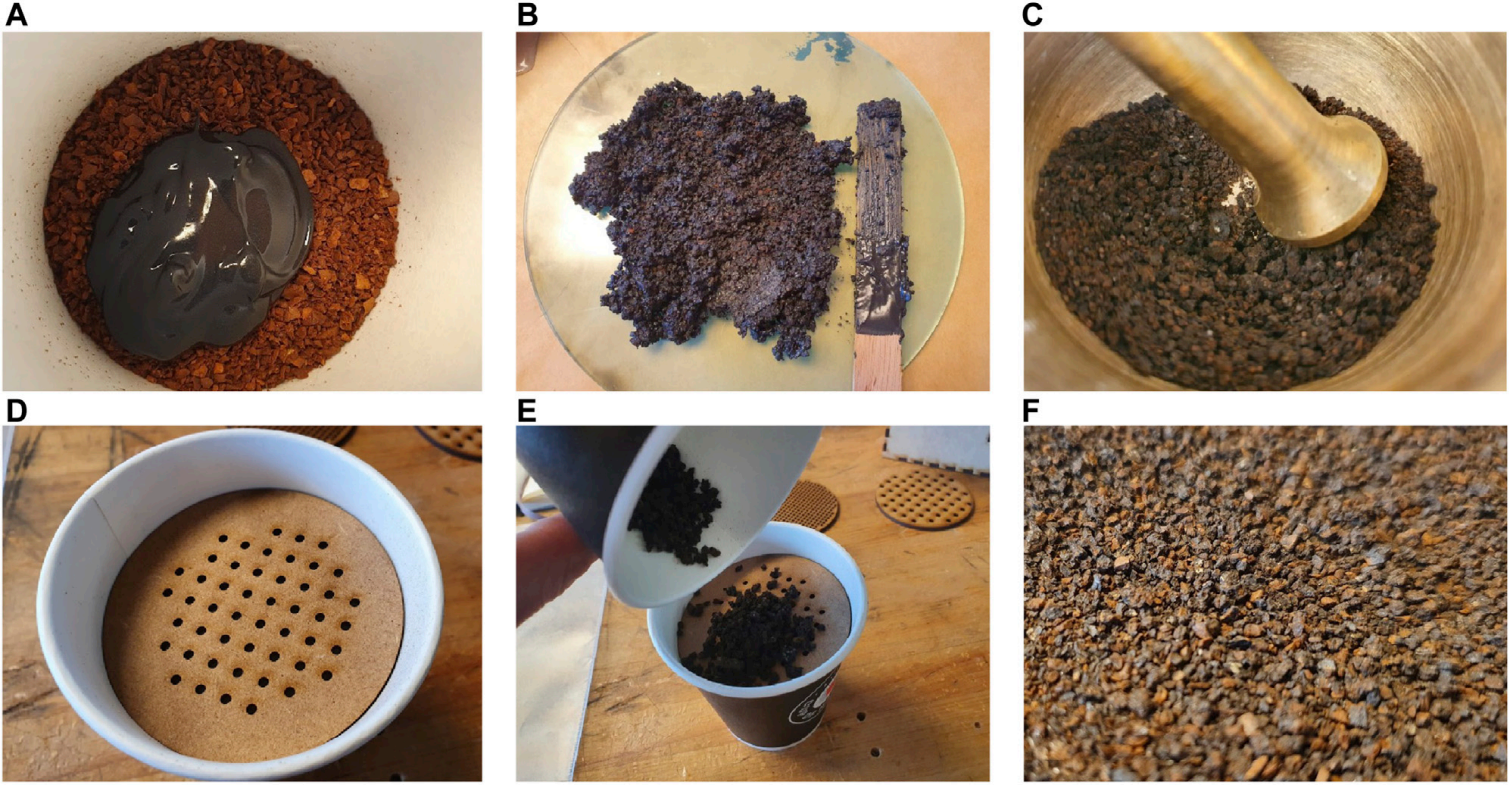
Manufacturing of magnetic granulate **(A)** 1:1 mixing ratio of coffee ground and magnetic paint **(B)** Consistency of the mixture **(C)** Grounding using mortar **(D)** and **(E)** Filtering **(F)** Finished result.

Ground coffee, which Putzu, Konstantinova, and Althoefer (2019) refer to as the gold standard within the field of granular jamming, was evaluated as the most viable option for our case. Ground coffee has been proven to be a successful granulate for jammers that need a large stiffness range (Brown et al., 2010; Cheng et al., 2012). The magnetic coffee ground was produced by mixing fine coffee ground and magnetic paint with a 1:1 volumetric ratio as seen in Figure 3 (Magnetic undercoat, Lefranc and Bourgeois Déco). After the mixture dried, it was ground to a size of approximately 2 mm using a mortar. Using a crushing technique, instead of grinding, produced less size dispersion of the granulate. Granules with a 1–2.4 mm size were filtered out with a perforated filter with circular holes (see Figures 3D,E). It is advantageous to use homogeneous monodisperse granules to make the output more repeatable (Genecov et al., 2014).

**FIGURE 4:**
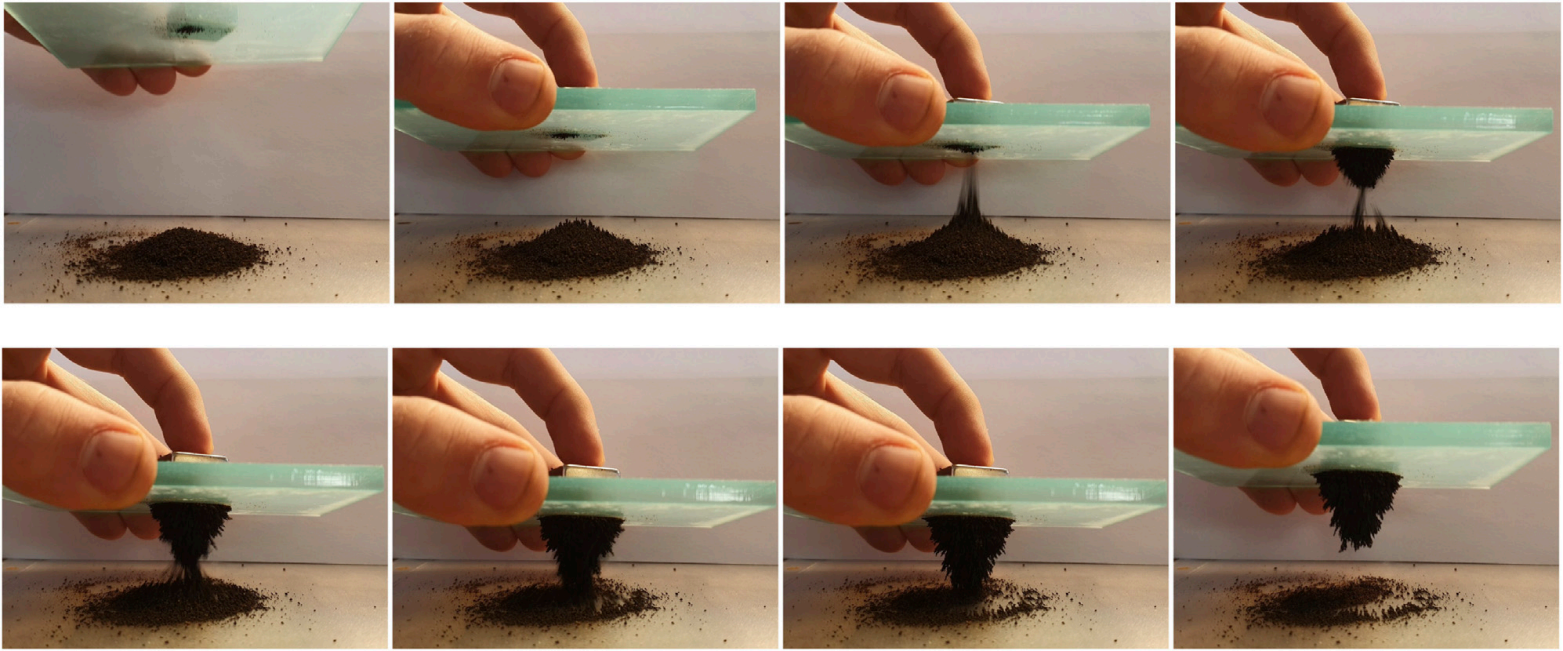
Manipulation of the magnetic coffee ground using a permanent magnet.

The manipulation of the ferromagnetic granulate using a permanent magnet is presented in Figure 4. The same type of spikes can be observed in both ferrofluids and iron shavings when in the presence of a magnetic field.

### 2.3 Chamber Design

Since the concept of this technology is different from traditional granular jamming, the choice of chamber material was evaluated on having surface friction that enabled the granules to be remotely manipulated inside the sealed chamber. Further, the material needed to be flexible to jam the particles together when a vacuum was applied. Different heat-sealing plastic types were evaluated, and a corrugated polyvinylchloride (PVC) film (0.2 mm for vacuum sealing applications) was deemed the most viable due to its flexibility and least warping lines. With the corrugated pattern, we avoided self-sealing as this was a problem with other materials.

### 2.4 Pneumatic Setup

**FIGURE 5:**
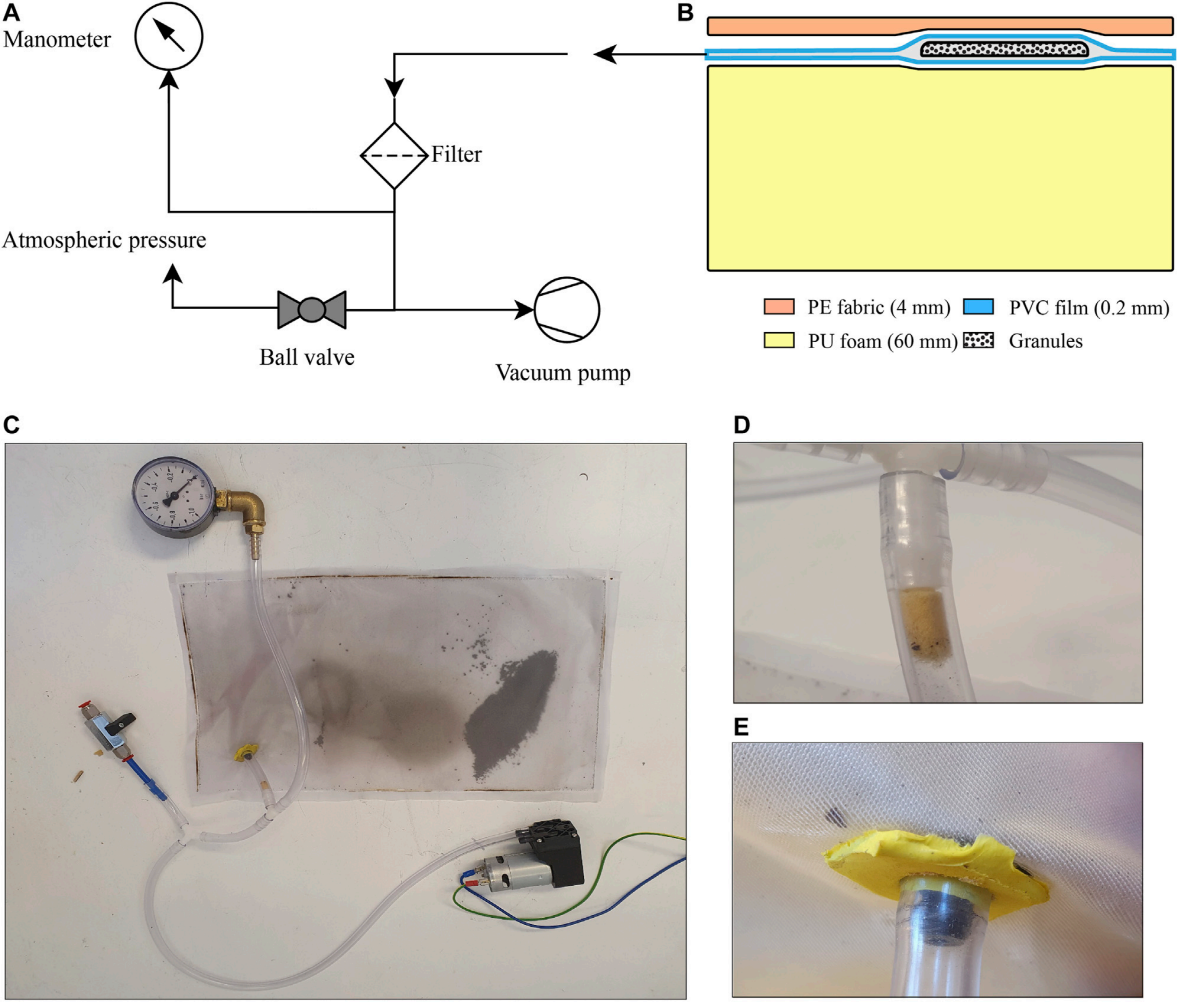
**(A)** Schematic presentation of the pneumatic setup **(B)** Palpation interface with layer material and thickness **(C)** The pneumatic setup **(D)** Filter **(E)** Inlet seal for vacuum chamber.

The pneumatic setup for the ferrogranular jamming concept is shown in Figure 5. The chamber is connected to the rest of the pneumatic system through a filter (Figure 5D). The 12 V vacuum pump (D2028B, SparkFun Electronics) delivers a vacuum level down to −0.54 bar. Next, a manometer is connected to measure the vacuum level. The vacuum pump is controlled using a speed controller. The chamber was made using an Impulse Heat Sealer (Audion Elektro Sealboy 235). A 3D-printed nozzle connects the chamber to the rest of the system, as seen in Figure 5E. Together with butyl vacuum sealant tape, it ensures minimal leakage at the inlet. A ball valve connects the system to atmospheric pressure when open.

## 3 Method: Experiment

A psychophysical experiment was designed to evaluate the functional abilities of the proposed concept by evaluating the user’s performance in locating and characterizing rendered irregularities. The experiment encompassed a palpation task, where qualitative and quantitative data were gathered on both participant performance and prototype reliability.

### 3.1 Experimental Test Setup

**FIGURE 6:**
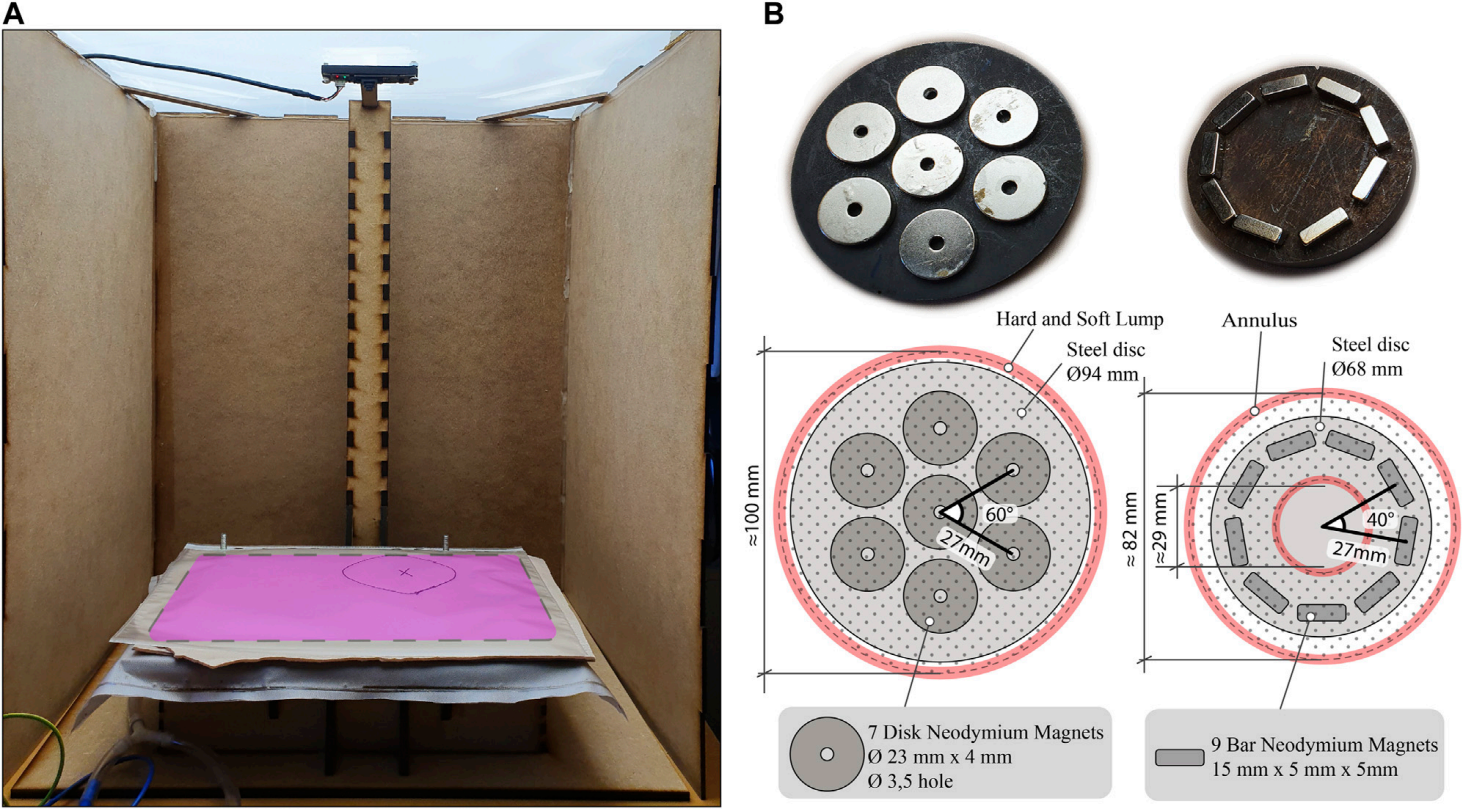
**(A)** Test rig with camera and cabinet setup (pink area is the palpable field) **(B)** Magnet arrangement with angular and radial distance shown. Approximated outlines for the generated outputs are also illustrated with measurements. (Left) Circular shape for Hard and Soft Lump (Right) Annulus shape with the hollow center.

The pneumatic system presented in 2.4 was integrated into the test cabinet shown in Figure 6A. A camera is fixed above the haptic interface. The cabinet walls ensure no bias from visual perception during the transition between conditions and provides a consistent working environment. In addition, an overhead LED panel eases picture processing by ensuring consistent lighting. The two different geometrical shapes were created with two arrangements of permanent neodymium magnets, as seen in Figure 6B. These magnets were held above the vacuum chamber, arranging the granules in the desired shape, before applying the vacuum. When vacuum was applied, the magnets could be removed and the granulate remained jammed in place. To alter the shape, or remove it, the vacuum was released, before the granules were manually dispersed, rearranged, or moved out of the palpable field. The structural parts of the test rig are laser-cut MDF. The palpable field (260 × 160 mm) is seen as the pink area in Figure 6A. We used 12 g of filtered ferromagnetic granulate in the chamber.

### 3.2 Experiment Design

All participants repeated the palpation task four times, under four different conditions. The irregularity could differ in hardness, position and form. The four conditions were as follows:

• C1: Baseline. No irregularity in the palpation field.
• C2: Annulus. Annular-shaped irregularity rendered with the magnet configuration seen in Figure 6B. Vacuum level: −0.4 to −0.6 bar, whereas −1 bar is a complete vacuum. Located in the lower left part of the field. Approximately 82 mm outer diameter and 29 mm inner diameter with area *M* = 4,915 mm^2^ *SD* = 371 mm^2^ *SE =* 70 mm^2^.
• C3: Hard Lump. A circular-shaped irregularity rendered with the magnet configuration seen in Figure 5B. Vacuum level: 0.4 to −0.6 bar. Located in the top right part of the field. Approximately 100 mm diameter with area *M* = 7,912 mm^2^ *SD* = 474 mm^2^ *SE =* 89 mm^2^.
• C4: Soft Lump. A circular-shaped irregularity rendered with the magnet configuration seen in Figure 5C. Vacuum level: 0.1 bar. Located in the top right part of the field. Approximately 100 mm diameter with area *M* = 8,094 mm^2^ *SD* = 641 mm^2^ *SE =* 121 mm^2^.

The sequence of the testing conditions was randomized to avoid potential learning or order effects. The order of conditions was also balanced, i.e., they appear the same number of times in each procedure step.

#### 3.2.1 Participants

N = 28 healthy engineering students were recruited to participate (21 male (75%) and 7 female (25%)). Twenty-seven participants were in the 21–29 years range and one participant in the 18–20 years range. None of the participants were trained in the test or had any relevant knowledge about the technology before participation. Participation was voluntary, and all gave informed consent to be part of the study.

#### 3.2.2 Experimental Procedure and Data Collection

**FIGURE 7:**
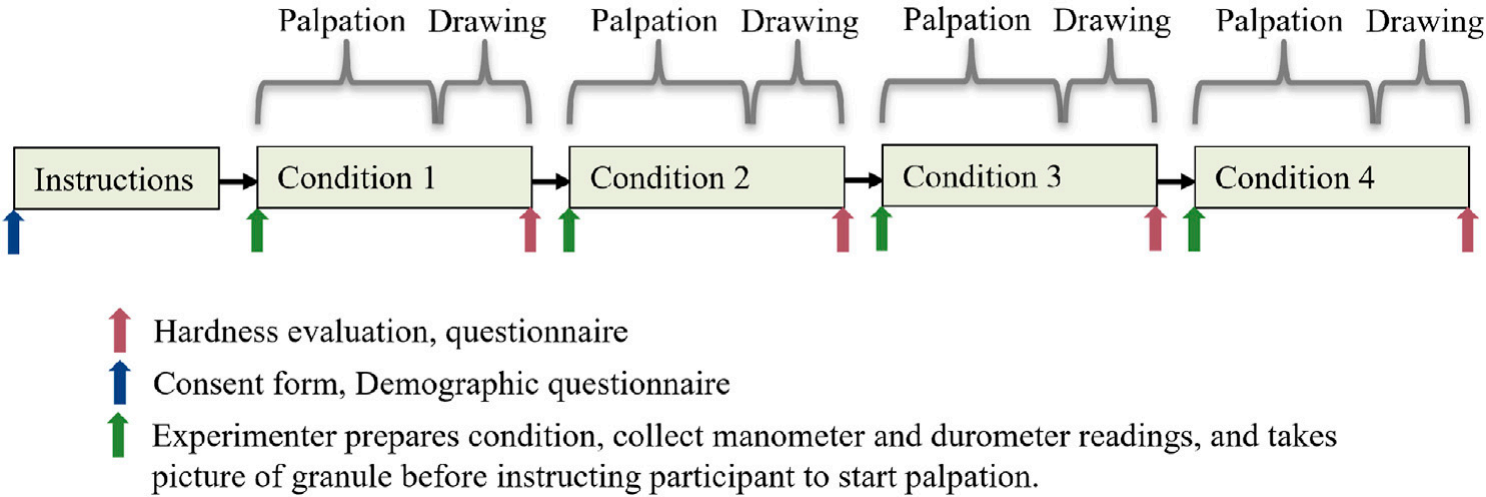
Experimental procedure timeline. The order of the conditions was randomized.

**FIGURE 8:**
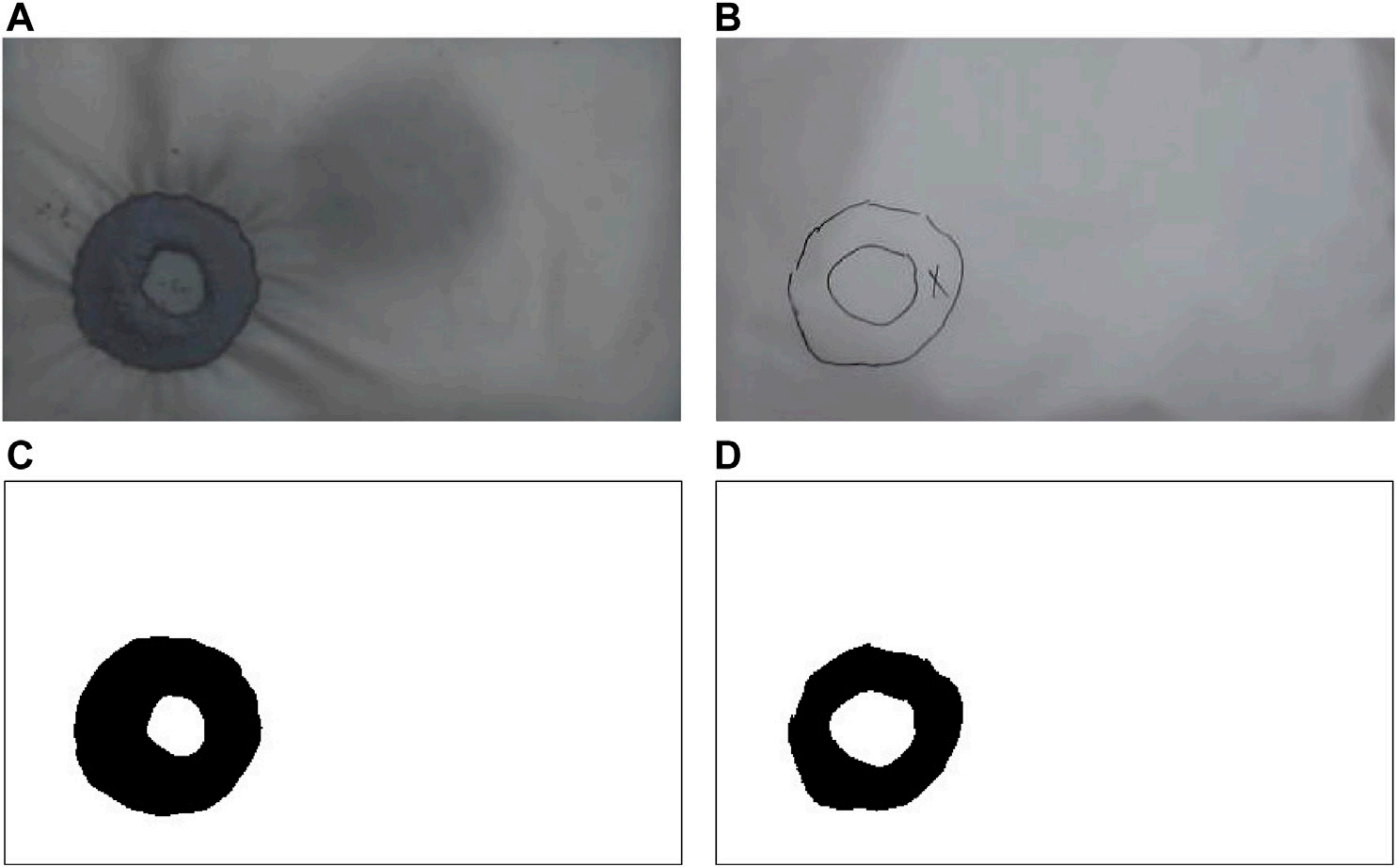
Unprocessed images of **(A)** Granulate and **(B)** Drawing. Binarized pictures of **(C)** Granulate and **(D)** Drawing.

The experimental procedure can be seen in Figure 7. After signing a consent form, the participants filled out a demographic questionnaire. Durometer and manometer readings and pictures of the granulate were sampled before the participant was seated in front of the test setup. The hardness of the irregularities was measured with a commercially available Shore durometer (Shenzhen Gairan Tech Co., X.F Type 00), following the requirements described in ISO 48–4:2018. A minimum of three measurements at different positions on the flat parts of the irregularity was performed. After objective data was collected, participants were instructed regarding the proceedings of the experiment. First, participants were told to palpate for a potential irregularity and say stop when they had control of the position and form. The participants did not get any instructions regarding technique to be used, other than using their hands to explore and feel for any irregularities in the field. We measured the time the participant used to find position and form of the irregularity. After completing each palpation, participants were asked to draw the contours of a possible irregularity on a sheet placed above the palpation field. More specifically, they were told to draw the outside and possible inside contours and put an x inside the area enclosing the proposedly identified irregularity (see Figure 8B). Pilot experiments showed that this instruction facilitated the participants who found an inside contour to also draw it, instead of drawing the outer contour only. Drawing data were captured using the camera.

To evaluate the hardness of the irregularity, a sampled selection of objects of varying hardness was used. These samples were numbered from 1 to 5 and had a Shore hardness of 00–20, 00–35, 00–55, 00–65, and 00–90, from soft to harder. The objects were presented similarly to the test setup using the same deformable backing and palpable cover as the palpation field. Thus, the participant could palpate the irregularity when doing the hardness test.

After each condition, participants reported their degree of agreement to a series of statements using a Likert Scale from 1 (Totally disagree) to 5 (Totally agree). The statements were: 1) It was hard to find the irregularity. 2) I am confident that I found the position and shape of the irregularity. 3) The irregularity had a constant/homogeneous hardness. To get a measure of a potential learning effect occurring during the experiments, participants also evaluated the statement: 4) I became better at finding the irregularity during the experiment., after completing the experiment.

### 3.3 Data Analysis

The data was collected throughout the experiment to answer the study’s research questions. Thus, experiment pictures were processed into binarized matrices that yielded objective data points describing irregularities’ and drawings’ respective positions and geometrical form. These data, together with the questionnaire and objective measurements, were statistically analyzed for reliability and differences between variables with SPSS Statistics (IBM SPSS Statistics 27, 2020).

#### 3.3.1 Picture Processing

Data about position and form was collected through images. The images of the granulate and drawings were then processed and analyzed using package OpenCV 4.5.1 in Python 3.0. The capturing code also took photos of the manometer during each test. The images were blurred before grayscaling and binarizing to remove noise. An adaptive Gaussian threshold was used on the pictures of the drawings to improve accuracy. The binarized results are shown in Figures 8C,D.

##### 3.3.1.1 Distance From Center to Center

The center point distance between granulate and drawings were calculated by finding the center of mass for both the granulate and the drawings using cv2.moments in Python. Then, the Euclidean distance ( ΔD ) was calculated between the two coordinates, using Eq. 1. x_1_ and y_1_ representing the coordinates for the granulate, while x_2_ and y_2_ representing the drawing.

$$\begin{array}{c} \Delta D=\sqrt{{\left(x_{1}-x_{2}\right)}^{2}+{\left(y_{1}-y_{2}\right)}^{2}\;} \end{array}$$ (1)

##### 3.3.1.2 Intersection Over Union

**FIGURE 9:**
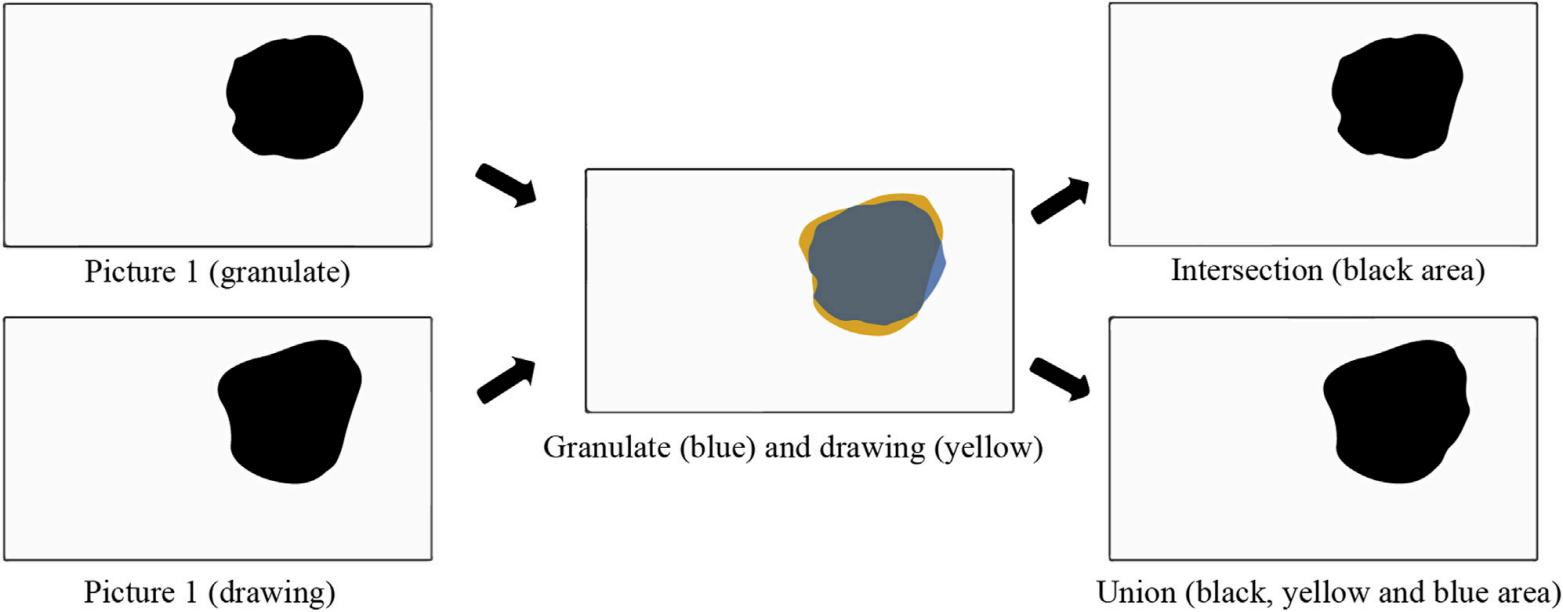
Visual presentation of intersection and union.

Intersection over Union (IoU) was used to evaluate the form. First, matrices of the intersection and union of the two binarized pictures were calculated using Python. Then, the number of black pixels (pixels with value 0) in the intersection was divided by the number of black pixels in the union, using Eq. 2. A visual representation of intersection and union is shown in Figure 9.

$$\begin{array}{c} IoU=\frac{Area\;of\;Intersection}{Area\;of\;Union} \end{array}$$ (2)

#### 3.4 Statistical Tests

To assess reliability, Fleiss’ kappa was ran to determine if there was an agreement between participants’ judgment of whether there was an irregularity or not (Lump or No Lump) in the four conditions. Fleiss’ kappa does not assume that the raters are identical for each condition (which is the case here), but this is the only test we know of that assesses the case when there are multiple raters. Therefore, we report this test along with the frequency. One-way repeated measures ANOVAs were used to investigate differences between conditions for continuous variables. Those were: IoU, hardness (durometer reading) and vacuum level (manometer reading). Assumptions regarding no outliers, normality, and sphericity were inspected with boxplots, histograms and Normal Q-Q Plots, and Mauchly’s Test of Sphericity. Violations of the outlier assumption were not removed since it only applied to Durometer and Manometer readings, which were used to corroborate that the conditions Hard Lump and Soft Lump differed in terms of hardness. In addition, a Friedman test was also conducted to ensure similar differences. A Greenhouse-Geisser correction was applied in the case of violating sphericity (Wickens and Keppel, 2004; Field, 2018). A Friedman test was used to investigate differences between conditions for discontinuous variables (the remaining variables), and in the case of more severe violations to ANOVA’s assumptions. Pairwise comparisons were performed with a Bonferroni correction for multiple comparisons for both ANOVA and Friedman. Some variables produced a statistically significant Friedman test, but without any significant pairwise comparisons. One reason might be the conservative nature of the multiple comparisons correction. An additional approach, multiple Wilcoxon signed-rank tests, was therefore used to follow up the Friedman tests. We deemed it acceptable to be less conservative since it is the first investigation of an early-stage prototype, and it was important to gain an understanding of where potential differences were. The Wilcoxon signed-rank tests was also used to obtain a z-score, used to estimate effect size (*r*) (Rosenthal, 1986; Field, 2018). For the ANOVAs, the sample effect size partial eta squared (η^2^), and population effect size partial omega squared (ω^2^) (Rosenthal, 1986) are reported. The significance level *p <* 0.05 was chosen for highly significant differences. *p–*values *≤* 0.10 were considered as interesting effects, again due to the experiment involving human participants evaluating an early-stage prototype. We believe a 10% probability for Type 1 error is acceptable in this case.

## 4 Results

Both objective and subjective data points were gathered throughout all four conditions described in 3.2.2. Each condition focused on localizing and characterizing a potential irregularity based on position, form and hardness. Additional descriptive statistics can be found in Supplementary Material.

### 4.1 Lump or No Lump: How Many Found an Irregularity?

In all three conditions with an irregularity (Annulus, Hard Lump and Soft Lump), all participants found an irregularity (100% agreement). In Baseline condition six participants (21.4%) found an irregularity, despite there not being one. The remaining 22 participants (78.6%) failed to find an irregularity. Fleiss’ kappa determined if there was an agreement between participants’ judgment of whether there was an irregularity or not (Lump or No Lump) in the four conditions. The agreement between participants’ judgements was statistically significant with κ= 0.723, 95% CI [0.722, 0.725], *p* < 0.001. The individual kappa’s for Lump and No Lump categories were also κ= 0.723, 95% CI [0.722, 0.725], *p* < 0.001. This statistic is the proportion of agreement over and above chance agreement, with 0 being no agreement and 1 being perfect agreement. An agreement of 0.723 can be classified as a good agreement (Landis and Koch, 1977).

As stated, six out of 28 participants found an irregularity in the baseline condition. Of these six, three participants drew contours with areas of 22, 64 and 147 mm^2^, which are small compared to the actual size of the irregularities. They are similar to granular remnants, which means they could be discarded as an error in the setup. Other participants commented on particle-sized irregularities in the Baseline condition but decided that they were not of sufficient size to be an actual irregularity. Removing these three participants results in three participants (12.0%) finding an irregularity in the Baseline condition, whereas 22 participants (88%) did not find an irregularity. Fleiss’ kappa was ran again with these three participants removed to investigate the magnitude of the potential error from the setup. The agreement between the remaining 25 participants was statistically significant with κ= 0.840, 95% CI [0.783, 0.896], *p* < 0.001. The individual kappa’s for Lump and No Lump categories were also κ= 0.840, 95% CI [0.783, 0.896], *p* < 0.001.

22 of 28 (78.57%) of the participants found the inner circle. In the two irregularity conditions 50 of 56 (89.29%) drawings were filled circles without any inner contour.

In summary, all participants agreed that there was an irregularity present in all irregularity conditions. Despite a few participants finding an irregularity where there was none, the overall agreement between participants was high.

### 4.2 Time

The users were not instructed to be as fast as possible but rather spend enough time to be sure of position and form of the irregularity. Therefore, the time represents the procedure time needed to find position and form of the irregularity to the best of the participant’s ability.

**FIGURE 10:**
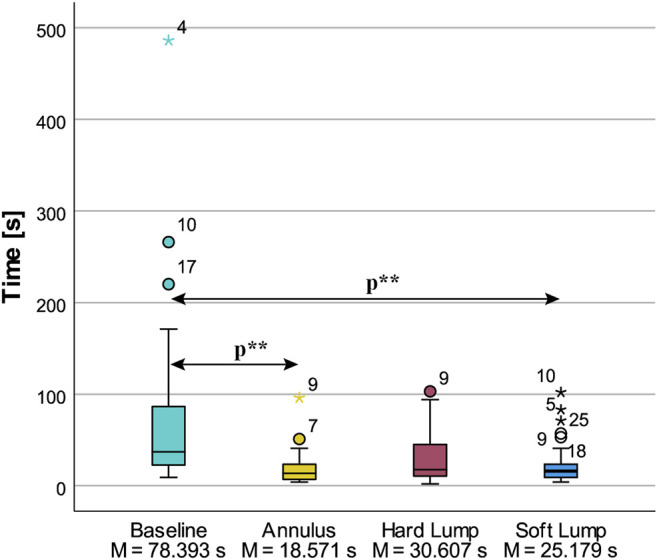
Descriptive statistics of procedure time to find the position and form of the irregularities. Statistically significant differences at *p* < 0.05 are indicated by p**.

Time was statistically significantly different in the four conditions, χ^2^(3) = 29.460, *p* < 0.001 as shown in Figure 10. Post hoc analysis revealed significant differences in Time from Baseline (Mdn = 37.0 s), 95% CI [25.0, 61.0] to Annulus (Mdn = 13.50 s), 95% CI [7.0, 21.0] (*p* < 0.001) and Soft Lump (Mdn = 16.00), 95% CI [11.0, 40.0] (*p* = 0.001) condition. It took longer to determine that there was no irregularity in Baseline condition, compared to finding it in Annulus and Soft Lump condition. The contrast comparing Annulus to Hard Lump (Mdn = 17.50), achieved a significance level *p =* 0.050 and effect size r = –0.50, and Hard Lump to Baseline had a significance level of *p =* 0.067 and effect size r = 0.48. We interpret this to be a notable difference. There was no significant difference between Annulus and Soft Lump (*p* = 0.80, r = –0.28) and Soft Lump and Hard Lump (*p* = 1.00, r = 0.22).

### 4.3 Position: Distance From Center to Center

**FIGURE 11:**
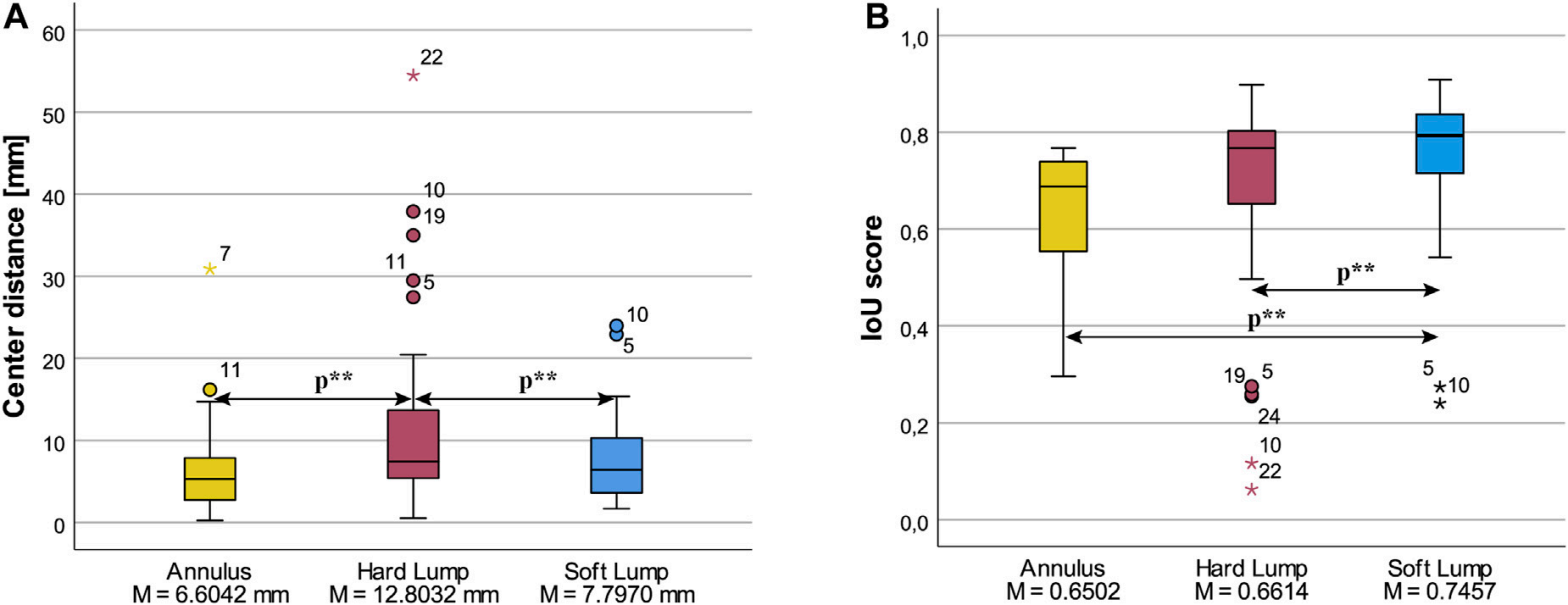
Box plots of **(A)** Center distance of granulate and drawing, and **(B)** Intersection over Union, for the three lump conditions. Statistically significant differences at *p* < 0.05 are indicated by p**.

The distance between the center of the irregularity to the center of the participants’ drawing was statistically significantly different in the three irregularity conditions, χ^2^ (2) = 16.357, *p* < 0.001. Post hoc analysis revealed statistically significant differences in center distance from Annulus (Mdn = 5.2920 mm), 95% CI [2.978, 7.097], to Hard Lump (Mdn = 7.4366 mm), 95% CI [6.331, 12.217] (*p* < 0.001), and from Soft Lump (Mdn = 6.3908 mm), 95% CI [3.836, 9.654], to Hard Lump condition (*Mdn =* 7.4366 mm) (*p* = 0.01). There was no significant difference in center distance between Annulus and Soft Lump (*p =* 1). We also observe that there was a greater spread in the Hard Lump condition. These results are plotted in Figure 11A.

### 4.4 Form: IoU

IoU was statistically significantly different in the three conditions, χ^2^ (2) = 12.071, *p* = 0.002. Post hoc pairwise comparisons with a Bonferroni correction for multiple comparisons yielded one significant difference between Soft Lump (Mdn = 0.793), 95% CI [0.728, 0.825], and Annulus (Mdn = 0.688), 95% CI [0.579, 0.735], *p =* 0.002, and a corrected *p =* 0.247 for both the Soft Lump vs Hard Lump (Mdn = 0.767), 95% CI [0.568, 0.754] comparison, and Hard Lump vs Annulus comparison (uncorrected p-value was *p =* 0.082). Post hoc Wilcoxon tests revealed a statistically significant difference between Annulus (Mdn = 0.688) and Soft Lump (Mdn = 0.793), *T* = 316.00, *p* = 0.010, r = 0.49, and a significant difference between Hard Lump (Mdn = 0.767), 95% CI [0.703, 0.0794], and Soft Lump (Mdn = 0.793), *T* = 304.00, *p* = 0. 021, *r* = 0.43. There was no difference between Annulus and Hard Lump, *T =* 263.00, *p =* 0. 021, *r =* 0.26 as plotted in Figure 11B.

### 4.5 Hardness

We compared perceived hardness, objective hardness measurements, and vacuum levels of the irregularity conditions.

#### 4.5.1 Perceived Hardness

**FIGURE 12:**
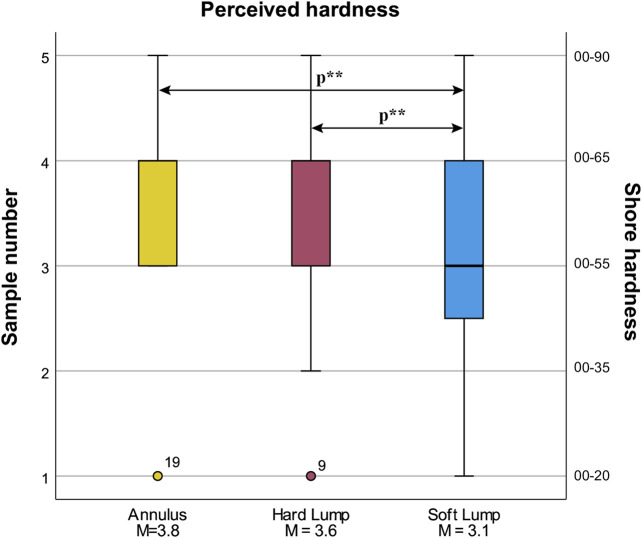
Box plot of perceived hardness (subjective) for the three lump conditions. Statistically significant differences at *p* < 0.05 are indicated by p**.

Perceived hardness was statistically significantly different in the three conditions, χ^2^ (2) = 9.129, *p* = 0.010. Post hoc pairwise comparisons with a Bonferroni correction for multiple comparisons yielded one significant difference between Soft Lump (Mdn = 3) and Annulus (Mdn = 4), *p =* 0.033, and a corrected *p =* 0.184 for the Soft Lump and Hard Lump comparison (uncorrected p-value was *p =* 0.061). Post hoc Wilcoxon tests revealed a statistically significant difference between Soft Lump (Mdn = 3) and Hard Lump (Mdn = 4), *T* = 132.00, *p* = 0. 032, *r* = –0.41, and a significant difference between Annulus (Mdn = 4) and Soft Lump (Mdn = 3), *T* = 55.00, *p* = 0.015, r = –0.46. There was no difference between Annulus and Hard Lump, *T =* 87.00, *p =* 0. 474, *r =* –0.14. These results are as expected. Participants perceived the hardness of Soft Lump to be less than that of both Hard Lump and Annulus which is shown in Figure 12.

#### 4.5.2 Durometric Measurements

**FIGURE 13:**
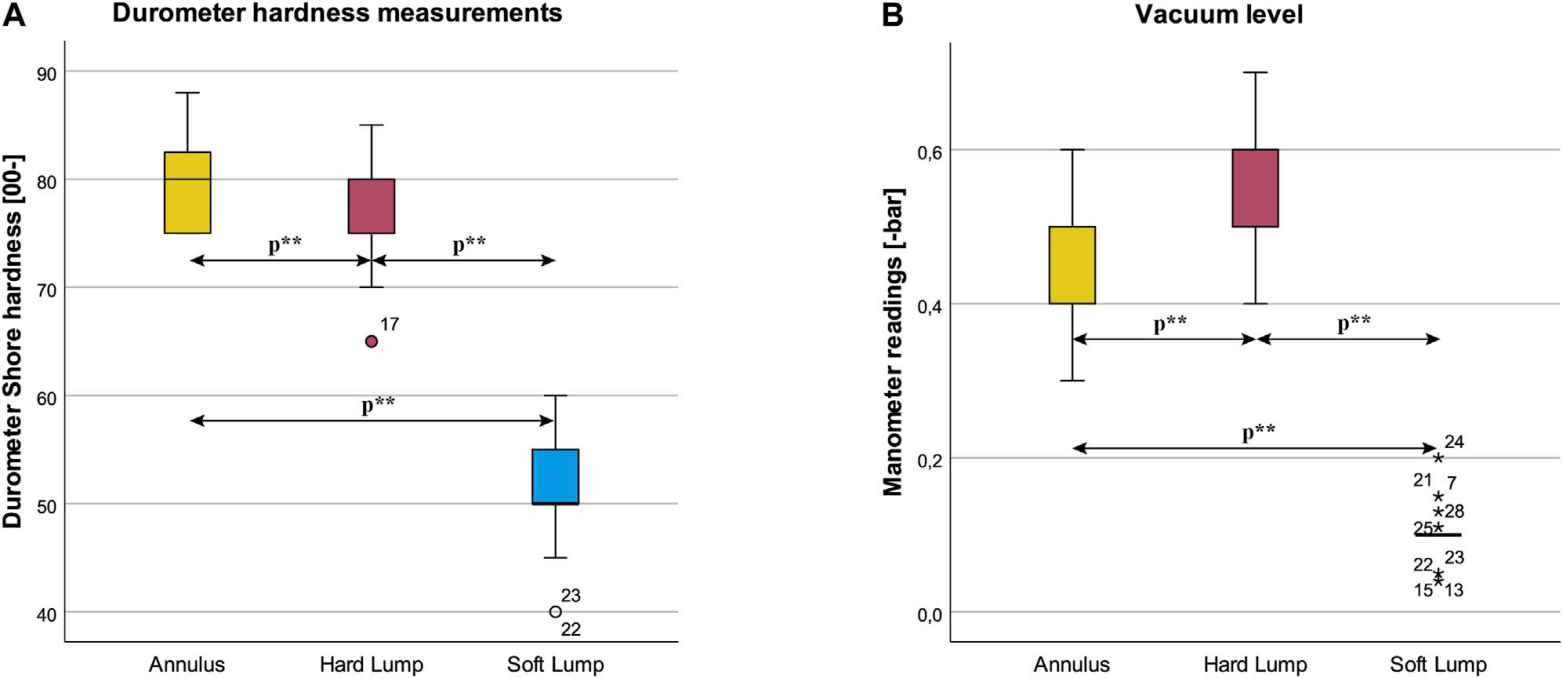
Box plots of **(A)** Durometer hardness measurements and **(B)** vacuum level for the three lump conditions. Statistically significant differences at *p* < 0.05 are indicated by p**.

There were 3 outliers as assessed by boxplot in Figure 13A. By visual inspection, the data was approximately normally distributed. The assumption of sphericity was violated, as assessed by Mauchly’s test of sphericity, χ^2^(2) = 6.825, *p* = 0.033. Therefore, a Greenhouse-Geisser correction was applied (ε = 0. 812). Results was statistically significant different in the three conditions F (1.625, 43.872) = 278.699, *p* < 0.001, η^2^ = 0.912, ω^2^ = 0.869. Durometric readings were: Annulus (*M =* 79.96), Hard Lump (*M =* 76.79), Soft Lump (*M =* 51.25). Post hoc analysis with a Bonferroni correction yielded statistically significantly difference between Annulus and Hard Lump (M = 3.179, 95% CI [0.63, 5.72], *p =* 0.011), between Annulus and Soft Lump (M = 0.28.714, 95% CI [24.71, 32.72], *p* < 0.001), and between Hard Lump and Soft Lump (M = 25.536, 95% CI [22.04, 0.29.03], *p* < 0.001).

#### 4.5.3 Manometer

There were several outliers as assessed by boxplot in Figure 13B. The data was approximately normally distributed by visual inspection. The assumption of sphericity was violated, as assessed by Mauchly’s test of sphericity, χ^2^(2) = 13.713, *p* = 0.001. Therefore, a Greenhouse-Geisser correction was applied (ε = 0. 709). Manometer was statistically significant different in the three conditions F (1.419, 38.301) = 228.636, *p* <0.001, η^2^ = 0.894, ω^2^ = 0.844. Manometer readings were: Annulus (*M =* 0.431), Hard Lump (*M =* 0.536), Soft Lump (*M =* 0.101). Post hoc analysis with a Bonferroni adjustment was statistically significantly different between Annulus and Hard Lump (M = –0.105, 95% CI [–0.17, –0.04], *p =* 0.002), between Annulus and Soft Lump (M = 0.330, 95% CI [0.29, 0.368], *p* < 0.001), and between Hard Lump and Soft Lump (M = 0.435, 95% CI [0.38, 0.49], *p* <0.001).

### 4.6 Questionnaire

Participants completed the questionnaire in the three irregularity conditions.

#### 4.6.1 How Hard Was It to Find the Position?

**FIGURE 14:**
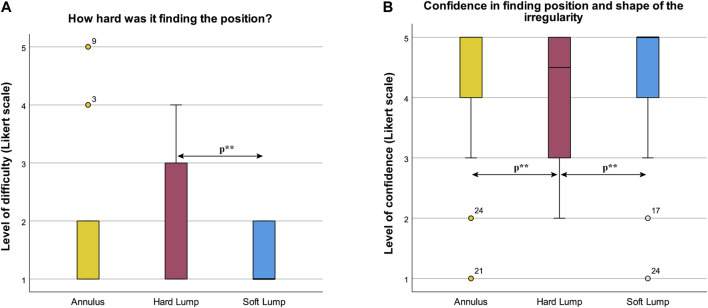
Box plot of results from the questionnaire **(A)** How hard was it finding the position, and **(B)** Self-assessment of confidence in the accuracy of their drawing.

Participants’ evaluation of how hard it was to find the irregularity was statistically significantly different in the three conditions, χ^2^(2) = 7.423, *p* = 0.024. Post hoc pairwise comparisons with a Bonferroni correction for multiple comparisons yielded no significant differences. Post hoc Wilcoxon tests revealed a statistically significant difference between Soft Lump (Mdn = 1) and Hard Lump (Mdn = 1), *T* = 63.00, *p* = 0.006, *r* = –0.52. There were no significant differences between Annulus (Mdn = 1) and Soft Lump (Mdn = 1), *T* = 27.00, *p* = 0.957, r = –0.01, or between Annulus and Hard Lump, *T =* 89.00, *p =* 0.096, *r =* –0.32. Participants found it hardest to locate the irregularity in the Hard Lump (see Figure 14A).

#### 4.6.2 Confidence in Finding Position and Shape of the Irregularity

Participants’ confidence in finding position and shape of the irregularity was statistically significantly different in the three irregularity conditions, χ^2^(2) = 8.926, *p* = 0.012. Post hoc pairwise comparisons with a Bonferroni correction for multiple comparisons yielded no significant differences. Post hoc Wilcoxon tests revealed a statistically significant decrease in confidence from Annulus (Mdn = 5) to Hard Lump (Mdn = 4.5), *T* = 12.00, *p* = 0.032, r = –0.40, and a significant decrease in confidence from Soft Lump (Mdn = 5) to Hard Lump (Mdn = 4.5), *T* = 13.50, *p* = 0.038, *r* = –0.39. There was no significant difference between Annulus and Soft Lump, *T* = 31.00, *p =* 0.276, *r =* –0.21. Participants were most confident in finding the position and shape of the irregularity in the Annulus and Soft Lump condition and less confident in finding the irregularity’s position and shape in the Hard Lump condition (see Figure 14B).

#### 4.6.3 Homogeneous Hardness

Participants’ evaluation of whether the irregularity had a constant/homogeneous hardness was not significantly different in the three irregularity conditions, χ^2^ (2) = 3.410, *p* = 0.182.

#### 4.6.4 Self-Assessed Improvement in Palpation

After completing the experiment, participants evaluated whether they thought they improved their palpation skills throughout the experiment. 20 participants (71.4%) thought they improved, 3 disagreed (10.7%), and 5 were neutral (17.9%).

## 5 Discussion

Introducing ferromagnetic granules in a jamming haptic interface has the quality to be a promising solution to produce larger tactile displays cheaply with high accuracy. Palpation trainers need to be robust, safe and have a high level of repeatability. Using adaptable palpation trainers increases the number of study cases and task-specific functionalities the trainer can accomplish. Thus, we think our concept can be taken further for use in a medical training equipment environment. Before that, however, there is a need for further development of the technology and contextual testing.

When comparing our data with relevant research (as mentioned in the Introduction), we have good results for people’s perception of both hardness and position. Bergmann Tiest and Kappers (2009) states that users are pretty good at determining hardness. Frisoli et al. (2011) states that cutaneous sensor modality is not affected by size, but kinesthetic performance is reduced with smaller-sized objects. To our knowledge, there is a lack of research on people’s perceptual exploration and characterization. Thus, this study could add to the body of knowledge concerning this aspect of both machine interaction and human tactile perception. The following section discusses the objective and subjective results gathered and how they answer our research questions. Further, an evaluation of the participant sampling is presented before we discuss the limitations and outlook of the study.

### 5.1 Interpreting Results

We defined that our haptic interface should be able to change the position, form and hardness of an irregularity recognizable by palpation. We chose a circle and an annulus as our two shapes to evaluate if people could locate and characterize them. A major part of the participants could differentiate between the circular lumps and the Annulus (78.57% recognized the inner circle of the Annulus, and 89.29% drew the circular lumps with no inner circle). Furthermore, all the participants found an irregularity in all conditions that had an irregularity. For the Baseline condition, six participants found a false positive. When determining the participants’ ability to describe the form, we used Intersection over Union (IoU). The median was promising for all three irregularity conditions, with the highest score for Soft Lump (0.793). From these three observations, we could conclude that the overall agreement between participants was great for form. Thus, our concept can manipulate the granules into different shapes that laypersons can distinguish by palpation.

However, an interesting result is that the IoU was significantly lower for Annulus than for Hard Lump and Soft Lump, while Annulus scored best at center point distance. A more logical assumption would be that IoU and center point distance is inversely proportional. There could be at least two reasons why we get a lower IoU score for Annulus. Initially, we observed from the participants contouring the Annulus that they struggled to get the size and position of the inner circle right. Due to how we calculated IoU, a wrong positioned hole yielded a more considerable difference in IoU than a similar error in outer contour. Also, the same error in the center point difference for Lump and Annulus gives a more significant change in IoU for Annulus because of the inner circle.

The results show a statistical difference for both objectively measured and perceived hardness between Soft lump and Hard Lump. Furthermore, when performing Wilcoxon tests on perceived hardness, there was a significantly lower value of the Soft Lump than the Hard Lump and Annulus condition. Thus, we have shown that participants can distinguish between hard and soft objects that the prototype produces, which is essential for palpation tasks, whereas characterizing the physical attributes such as form and hardness of identified irregularities is essential.

Considering how participants conducted the palpation tasks, time spent is of interest. The five most extended procedure times were on baseline condition, and three of them had baseline as the first condition. For example, participant No. 4 stated that there was no irregularity after 90 s before spending six more minutes to palpate before finding a false positive. No. 21 expressed hesitancy after 1 minute, and then spent two more minutes palpating before concluding with a true negative. No. 17 expressed insecurity before spending 2–3 more minutes searching, ending with a true negative. From the respective participant’s confidence data, the participant with the false positive (no. 4) answer a four on the Likert scale, i.e., partially agreeing that they were sure they found the correct position and form of the irregularity. Also, all three participants had good results in all irregularity conditions. This connection could mean that some people struggle to trust their sense of touch. The baseline condition presents ambiguity as there is nothing to palpate, and we believe having it as the first condition increased ambiguity and thus uncertainty in participants who probably expected an irregularity.

Another aspect is the repeatability of our testing equipment. We tried to develop a haptic interface that can alter and maintain hardness, position and form of palpable outputs with high repeatability. However, while prototyping the granular jammer, it became apparent that repeatedly creating geometries with identical shape and hardness was challenging. While the outline for the shapes varied for each sample as a result of the manual setup and granule dispersion, the gathered images showed only a small deviation of rendered area for each irregularity condition. The durometric measurements did, however, show a wide hardness range within a prepared condition. This inhomogeneous hardness from granular jamming is similar to the findings of Genecov, Stanley, and Okamura (2014). We thought of two reasons for this, firstly, how the hardness is highly dependent on how the granules interlock or position themselves across the irregularity. Secondly, because the arrangement of granules was made manually, there was an unavoidable variation in the produced output geometries and thus granulate concentration across the area.

Considering the pressure readings for the setup, Soft Lump had more outliers as a result of the vacuum level being manually set (and adjusted). Compared to Hard Lump and Annulus which had a hard stop, governed by the maximum vacuum the setup could provide. Given these being different geometries thus yielding different volumes to drain the air from, this could cause the difference in obtained vacuum level. However, our results from the perceived hardness showed no significant difference between Annulus and Hard Lump. As this being a first prototype, challenges concerning repeatability is expected and the overall results show great promise for this concept to be improved further to address these limitations.

When looking at the questionnaire data, confidence was highest in finding the position and shape of the Annulus and Soft Lump condition. We expected that the Hard Lump would be the easiest to find due to a sharper edge and thus greater difference between the Hard Lump and the palpable area. However, this was not the case. In retrospect we suspect this was due to the increased vacuum level instantly jamming the granules not allowing them to evenly distribute and conform to a smooth shape. This could cause the edges of the Hard Lump to be more jagged/rivet than the edge of the Soft Lump, which had a more circular shape in comparison. We therefore believe this might have made participants more uncertain of where the edge was. Moreover, since the edge of the Hard Lump varied more compared to a circle, this may have contributed to that it was more difficult to find the center of it.

Participants reported they got better at finding the irregularity throughout the experiment, meaning it could be used as a training device for palpation exercises. However, a reported high level of confidence and low level of difficulty for each condition could mean the task being too easy to perform, not leaving much room for progression and learning. Given the ability to find and characterize the irregularities sufficiently, and having a high confidence in doing so, further steps should be made to tailor level of difficulty to specific scenarios and investigating the use of the device in a medical context. The concept has been experimentally, shown to facilitate users’ palpation skills by speed, location, shape, and hardness differentiation of palpable findings. Other learning objectives could involve motoric technique and following procedural algorithms, which should be explored in further development of the concept.

### 5.2 Participant Sampling

In this experiment, the 28 participants were all engineering students who did not have any previous experience with palpation as a medical examination technique. The participants did not get any technical or strategic instructions, meaning their palpation approach would be different to a medical professional. Therefore, we have shown that our concept works for presenting generic geometric shapes for laypersons, which is a promising result considering this a training device. Moreover, having participants with prior medical experience, would thus require a higher level of difficulty. A sample size of 28 was adequate compared to similar studies (Gerling et al., 2003; Asgar-Deen et al., 2020). Also, as we got statistically significant differences between relevant data points, such as the hardness of Soft Lump and Hard Lump, more participants would most likely not produce other results. However, a higher sample size would reduce the possibility of an accepted hypothesis being incorrect.

### 5.3 Limitations and Further Work

In this research, sensing, automation or participant feedback has not been addressed nor implemented in the palpation concept. The prototype and subsequently the experiments with the prototype are not tied to a medical context. Instead, it explores some of the capabilities and extreme conditions the haptic device can output. Hence, it has not been within the scope of this research to model, synthesize, or simulate physiological attributes for palpation. Nevertheless, we lack to prove that our haptic interface is helpful in medical training because of a simplified experiment focusing on planar perceptual exploration. Therefore, in further development, more levels of difficulty, complex geometries, hardness profiles, locations, and dynamic abilities should be explored. As palpation tasks seldom concerns irregularities in one plane, investigating multiple jamming layers, or simulating depth of palpation by dynamic stiffness control should be investigated. A positive backpressure in combination with ferrogranular jamming could yield 3D-shapes with high tactile resolution and geometrical freedom (Koehler et al., 2020; He et al., 2021). In further work, we seek to test the concept with users who can provide feedback and evaluation on a medical basis. This could reveal hitherto unexplored concept potentials and critical functions to pursue.

## 6 Conclusion

This work has described the development and testing of a novel haptic interface concept that uses ferrogranular jamming. This concept was developed as a compliant simulation interface for medical palpation training, with the ability to simulate geometrical objects of various shapes and tactile properties. The concept was tested by having 28 untrained participants perform a set of structured palpation tasks in an experiment. The experiment consisted of four conditions, one baseline and three containing a palpable irregularity. These conditions were chosen to evaluate if the interface could produce various shapes and hardness levels, while also investigating participants’ palpation skills. Given the results of the experiment, we conclude that the concept can create palpable objects with variable hardness by adjusting the jamming vacuum. Laypersons can distinguish these objects by palpation, both by the hardness, location, and shape of objects with good accuracy. Thus, this study also provides insights on peoples’ perceptual abilities in explorative palpation. It shows the ability to locate and characterize palpable objects of varying shape and hardness in a satisfactory manner. Further, the results show that, the task was not considered very challenging. This combined with participants reported high level of confidence in performance, indicates that increased difficulty might be required to ensure room for improvement and learning. However, as participants also reported improvements in their palpation skills during the experiment, the technology looks promising to be further developed for medical training applications.

Considering this being an early conceptual prototype, this study revealed opportunities and challenges yet to be addressed. In further work, we want to explore whether the interface can be used as a palpation tool in medical simulation by qualitative testing with expert users. This will require palpable objects where both hardness, shape, and difficulty are tailored to the medical scenarios we want to simulate. Other technical aspects of the ferrogranular jamming concept we want to explore are sensing and feedback, automation, and dynamic and responsive tactile abilities. Collectively, this could improve the experience of using this technology in simulation-based medical training.

## Data Availability

The raw data supporting the conclusions of this article is available in the following repository. SBR; MA; HD; and MS, 2021, Replication Data for: Perception by Palpation: Development and Testing of a Haptic Ferrogranular Jamming Surface, https://doi.org/10.18710/OCMXVP, DataverseNO, V1.
