# Supplementary material for: Perception by Palpation: Development and Testing of a Haptic Ferrogranular Jamming Surface
Source: Front Robot AI. 2021 Sep 28;8:745234. doi: 10.3389/frobt.2021.745234 (PMC8505531; doi:10.3389/frobt.2021.745234)
Supplement: Supplementary file 1 [file DataSheet1.docx]

Supplementary Materials

# Supplementary Data and Code

The data and code underlying this study is deposited in the following repository:

Rørvik, Sigurd Bjarne; Auflem, Marius; Dybvik, Henrikke; Steinert, Martin, 2021, "Replication Data for: Perception by Palpation: Development and Testing of a Haptic Ferrogranular Jamming Surface", <https://doi.org/10.18710/OCMXVP>, DataverseNO, V1

# Supplementary Descriptive Statistics

## Time

Table 1 Time [s]

| *Descriptives* | | | | |
| --- | --- | --- | --- | --- |
| Condition | | | Statistic | Std. Error |
| Baseline | Mean | | 78.3929 | 19.51687 |
|  | 95% Confidence Interval for Mean | Lower Bound | 38.3475 |  |
|  |  | Upper Bound | 118.4382 |  |
|  | 5% Trimmed Mean | | 63.0794 |  |
|  | Median | | 37.0000 |  |
|  | Variance | | 10665.433 |  |
|  | Std. Deviation | | 103.27358 |  |
|  | Minimum | | 9.00 |  |
|  | Maximum | | 486.00 |  |
|  | Range | | 477.00 |  |
|  | Interquartile Range | | 65.00 |  |
|  | Skewness | | 2.734 | .441 |
|  | Kurtosis | | 8.638 | .858 |
| Annulus | Mean | | 18.5714 | 3.61816 |
|  | 95% Confidence Interval for Mean | Lower Bound | 11.1476 |  |
|  |  | Upper Bound | 25.9953 |  |
|  | 5% Trimmed Mean | | 15.7937 |  |
|  | Median | | 13.5000 |  |
|  | Variance | | 366.550 |  |
|  | Std. Deviation | | 19.14550 |  |
|  | Minimum | | 4.00 |  |
|  | Maximum | | 96.00 |  |
|  | Range | | 92.00 |  |
|  | Interquartile Range | | 17.25 |  |
|  | Skewness | | 2.775 | .441 |
|  | Kurtosis | | 9.599 | .858 |
| Hard Lump | Mean | | 30.6071 | 5.44801 |
|  | 95% Confidence Interval for Mean | Lower Bound | 19.4288 |  |
|  |  | Upper Bound | 41.7855 |  |
|  | 5% Trimmed Mean | | 28.2222 |  |
|  | Median | | 17.5000 |  |
|  | Variance | | 831.062 |  |
|  | Std. Deviation | | 28.82815 |  |
|  | Minimum | | 2.00 |  |
|  | Maximum | | 103.00 |  |
|  | Range | | 101.00 |  |
|  | Interquartile Range | | 35.25 |  |
|  | Skewness | | 1.349 | .441 |
|  | Kurtosis | | .679 | .858 |
| Soft Lump | Mean | | 25.1786 | 4.76846 |
|  | 95% Confidence Interval for Mean | Lower Bound | 15.3945 |  |
|  |  | Upper Bound | 34.9626 |  |
|  | 5% Trimmed Mean | | 22.3571 |  |
|  | Median | | 16.0000 |  |
|  | Variance | | 636.671 |  |
|  | Std. Deviation | | 25.23233 |  |
|  | Minimum | | 4.00 |  |
|  | Maximum | | 102.00 |  |
|  | Range | | 98.00 |  |
|  | Interquartile Range | | 14.75 |  |
|  | Skewness | | 1.834 | .441 |
|  | Kurtosis | | 2.703 | .858 |

## Position: Distance from Center to Center

Table 2 Distance from Center to Center [mm]

| *Descriptives* | | | | |
| --- | --- | --- | --- | --- |
| Condition | | | Statistic | Std. Error |
| Annulus | Mean | | 6.6042 | 1.17907 |
|  | 95% Confidence Interval for Mean | Lower Bound | 4.1850 |  |
|  |  | Upper Bound | 9.0234 |  |
|  | 5% Trimmed Mean | | 5.8315 |  |
|  | Median | | 5.2920 |  |
|  | Variance | | 38.926 |  |
|  | Std. Deviation | | 6.23903 |  |
|  | Minimum | | .23 |  |
|  | Maximum | | 30.89 |  |
|  | Range | | 30.66 |  |
|  | Interquartile Range | | 5.51 |  |
|  | Skewness | | 2.443 | .441 |
|  | Kurtosis | | 7.851 | .858 |
| Hard Lump | Mean | | 12.8032 | 2.41790 |
|  | 95% Confidence Interval for Mean | Lower Bound | 7.8420 |  |
|  |  | Upper Bound | 17.7643 |  |
|  | 5% Trimmed Mean | | 11.4242 |  |
|  | Median | | 7.4366 |  |
|  | Variance | | 163.695 |  |
|  | Std. Deviation | | 12.79433 |  |
|  | Minimum | | .49 |  |
|  | Maximum | | 54.48 |  |
|  | Range | | 53.99 |  |
|  | Interquartile Range | | 8.82 |  |
|  | Skewness | | 1.878 | .441 |
|  | Kurtosis | | 3.303 | .858 |
| Soft Lump | Mean | | 7.7970 | 1.08712 |
|  | 95% Confidence Interval for Mean | Lower Bound | 5.5664 |  |
|  |  | Upper Bound | 10.0276 |  |
|  | 5% Trimmed Mean | | 7.2522 |  |
|  | Median | | 6.3908 |  |
|  | Variance | | 33.091 |  |
|  | Std. Deviation | | 5.75249 |  |
|  | Minimum | | 1.68 |  |
|  | Maximum | | 23.96 |  |
|  | Range | | 22.29 |  |
|  | Interquartile Range | | 6.94 |  |
|  | Skewness | | 1.459 | .441 |
|  | Kurtosis | | 2.188 | .858 |

## Form: IoU

Table 3 IoU [overlap in percentage]

| *Descriptives* | | | | |
| --- | --- | --- | --- | --- |
| Condition | | | Statistic | Std. Error |
| Annulus | Mean | | .650150 | .0223059 |
|  | 95% Confidence Interval for Mean | Lower Bound | .604382 |  |
|  |  | Upper Bound | .695918 |  |
|  | 5% Trimmed Mean | | .661164 |  |
|  | Median | | .687700 |  |
|  | Variance | | .014 |  |
|  | Std. Deviation | | .1180318 |  |
|  | Minimum | | .2956 |  |
|  | Maximum | | .7671 |  |
|  | Range | | .4715 |  |
|  | Interquartile Range | | .1911 |  |
|  | Skewness | | -1.305 | .441 |
|  | Kurtosis | | 1.492 | .858 |
| Hard Lump | Mean | | .661350 | .0451526 |
|  | 95% Confidence Interval for Mean | Lower Bound | .568705 |  |
|  |  | Upper Bound | .753995 |  |
|  | 5% Trimmed Mean | | .680763 |  |
|  | Median | | .767400 |  |
|  | Variance | | .057 |  |
|  | Std. Deviation | | .2389249 |  |
|  | Minimum | | .0622 |  |
|  | Maximum | | .8980 |  |
|  | Range | | .8358 |  |
|  | Interquartile Range | | .1681 |  |
|  | Skewness | | -1.486 | .441 |
|  | Kurtosis | | .973 | .858 |
| Soft Lump | Mean | | .745721 | .0315139 |
|  | 95% Confidence Interval for Mean | Lower Bound | .681060 |  |
|  |  | Upper Bound | .810383 |  |
|  | 5% Trimmed Mean | | .764310 |  |
|  | Median | | .792750 |  |
|  | Variance | | .028 |  |
|  | Std. Deviation | | .1667557 |  |
|  | Minimum | | .2395 |  |
|  | Maximum | | .9086 |  |
|  | Range | | .6691 |  |
|  | Interquartile Range | | .1261 |  |
|  | Skewness | | -1.980 | .441 |
|  | Kurtosis | | 3.932 | .858 |

## Hardness

### Perceived Hardness

To evaluate the hardness of the irregularity, a sampled selection of objects of varying hardness was used, with Shore hardness 00-20, 00-35, 00-55, 00-65, and 00-90, whereas 00-90 is hardest.. The ordinal encoding was 1 = 00-20, 2 = 00-35, 3 = 00-55, 4 = 00-65, and 5 = 00-90.

Table 4 Perceived Hardness

| *Descriptives* | | | | |
| --- | --- | --- | --- | --- |
| Condition | | | Statistic | Std. Error |
| Annulus | Mean | | 3.75 | .151 |
|  | 95% Confidence Interval for Mean | Lower Bound | 3.44 |  |
|  |  | Upper Bound | 4.06 |  |
|  | 5% Trimmed Mean | | 3.80 |  |
|  | Median | | 4.00 |  |
|  | Variance | | .639 |  |
|  | Std. Deviation | | .799 |  |
|  | Minimum | | 1 |  |
|  | Maximum | | 5 |  |
|  | Range | | 4 |  |
|  | Interquartile Range | | 1 |  |
|  | Skewness | | -1.377 | .441 |
|  | Kurtosis | | 4.207 | .858 |
| Hard Lump | Mean | | 3.57 | .174 |
|  | 95% Confidence Interval for Mean | Lower Bound | 3.21 |  |
|  |  | Upper Bound | 3.93 |  |
|  | 5% Trimmed Mean | | 3.62 |  |
|  | Median | | 4.00 |  |
|  | Variance | | .847 |  |
|  | Std. Deviation | | .920 |  |
|  | Minimum | | 1 |  |
|  | Maximum | | 5 |  |
|  | Range | | 4 |  |
|  | Interquartile Range | | 1 |  |
|  | Skewness | | -.840 | .441 |
|  | Kurtosis | | 1.138 | .858 |
| Soft Lump | Mean | | 3.14 | .190 |
|  | 95% Confidence Interval for Mean | Lower Bound | 2.75 |  |
|  |  | Upper Bound | 3.53 |  |
|  | 5% Trimmed Mean | | 3.17 |  |
|  | Median | | 3.00 |  |
|  | Variance | | 1.016 |  |
|  | Std. Deviation | | 1.008 |  |
|  | Minimum | | 1 |  |
|  | Maximum | | 5 |  |
|  | Range | | 4 |  |
|  | Interquartile Range | | 2 |  |
|  | Skewness | | -.539 | .441 |
|  | Kurtosis | | -.292 | .858 |

### Durometric Measurements

3 measurements were made for each condition, and averaged in increments of 5, e.g., 70, 75, 80.

Table 5 Durometric [Shore hardness in 00-scale]

| *Descriptives* | | | | |
| --- | --- | --- | --- | --- |
| Condtion | | | Statistic | Std. Error |
| Annulus | Mean | | 79.96 | .750 |
|  | 95% Confidence Interval for Mean | Lower Bound | 78.43 |  |
|  |  | Upper Bound | 81.50 |  |
|  | 5% Trimmed Mean | | 79.83 |  |
|  | Median | | 80.00 |  |
|  | Variance | | 15.739 |  |
|  | Std. Deviation | | 3.967 |  |
|  | Minimum | | 75 |  |
|  | Maximum | | 88 |  |
|  | Range | | 13 |  |
|  | Interquartile Range | | 9 |  |
|  | Skewness | | .255 | .441 |
|  | Kurtosis | | -.830 | .858 |
| Hard Lump | Mean | | 76.79 | .822 |
|  | 95% Confidence Interval for Mean | Lower Bound | 75.10 |  |
|  |  | Upper Bound | 78.47 |  |
|  | 5% Trimmed Mean | | 76.90 |  |
|  | Median | | 75.00 |  |
|  | Variance | | 18.915 |  |
|  | Std. Deviation | | 4.349 |  |
|  | Minimum | | 65 |  |
|  | Maximum | | 85 |  |
|  | Range | | 20 |  |
|  | Interquartile Range | | 5 |  |
|  | Skewness | | -.434 | .441 |
|  | Kurtosis | | 1.011 | .858 |
| Soft Lump | Mean | | 51.25 | 1.080 |
|  | 95% Confidence Interval for Mean | Lower Bound | 49.03 |  |
|  |  | Upper Bound | 53.47 |  |
|  | 5% Trimmed Mean | | 51.39 |  |
|  | Median | | 50.00 |  |
|  | Variance | | 32.639 |  |
|  | Std. Deviation | | 5.713 |  |
|  | Minimum | | 40 |  |
|  | Maximum | | 60 |  |
|  | Range | | 20 |  |
|  | Interquartile Range | | 5 |  |
|  | Skewness | | -.050 | .441 |
|  | Kurtosis | | -.391 | .858 |

### Manometer

The manometer used measured vacuum on a scale from 0 to -1 bar. Absolute values were noted.

Table 6 Manometer [bar]

| *Descriptives* | | | | |
| --- | --- | --- | --- | --- |
|  | | | Statistic | Std. Error |
| Annulus | Mean | | .4307 | .01619 |
|  | 95% Confidence Interval for Mean | Lower Bound | .3975 |  |
|  |  | Upper Bound | .4639 |  |
|  | 5% Trimmed Mean | | .4286 |  |
|  | Median | | .4000 |  |
|  | Variance | | .007 |  |
|  | Std. Deviation | | .08568 |  |
|  | Minimum | | .30 |  |
|  | Maximum | | .60 |  |
|  | Range | | .30 |  |
|  | Interquartile Range | | .10 |  |
|  | Skewness | | .516 | .441 |
|  | Kurtosis | | -.246 | .858 |
| Hard Lump | Mean | | .5361 | .01954 |
|  | 95% Confidence Interval for Mean | Lower Bound | .4960 |  |
|  |  | Upper Bound | .5762 |  |
|  | 5% Trimmed Mean | | .5345 |  |
|  | Median | | .5000 |  |
|  | Variance | | .011 |  |
|  | Std. Deviation | | .10340 |  |
|  | Minimum | | .40 |  |
|  | Maximum | | .70 |  |
|  | Range | | .30 |  |
|  | Interquartile Range | | .10 |  |
|  | Skewness | | .323 | .441 |
|  | Kurtosis | | -.968 | .858 |
| Soft Lump | Mean | | .1011 | .00598 |
|  | 95% Confidence Interval for Mean | Lower Bound | .0888 |  |
|  |  | Upper Bound | .1133 |  |
|  | 5% Trimmed Mean | | .0996 |  |
|  | Median | | .1000 |  |
|  | Variance | | .001 |  |
|  | Std. Deviation | | .03166 |  |
|  | Minimum | | .04 |  |
|  | Maximum | | .20 |  |
|  | Range | | .16 |  |
|  | Interquartile Range | | .00 |  |
|  | Skewness | | .754 | .441 |
|  | Kurtosis | | 3.185 | .858 |

## Questionnaire

### How Hard was it to Find the Position?

Participants reported their degree of agreement to a statement using a Likert Scale from 1 (Totally disagree) to 5 (Totally agree). The statement was: It was hard to find the irregularity.

| *Descriptives* | | | | |
| --- | --- | --- | --- | --- |
|  | | | Statistic | Std. Error |
| Hard_Finding_Position_Annulus | Mean | | 1.46 | .182 |
|  | 95% Confidence Interval for Mean | Lower Bound | 1.09 |  |
|  |  | Upper Bound | 1.84 |  |
|  | 5% Trimmed Mean | | 1.31 |  |
|  | Median | | 1.00 |  |
|  | Variance | | .925 |  |
|  | Std. Deviation | | .962 |  |
|  | Minimum | | 1 |  |
|  | Maximum | | 5 |  |
|  | Range | | 4 |  |
|  | Interquartile Range | | 1 |  |
|  | Skewness | | 2.666 | .441 |
|  | Kurtosis | | 7.408 | .858 |
| Hard_Finding_Position_Hard Lump | Mean | | 1.93 | .224 |
|  | 95% Confidence Interval for Mean | Lower Bound | 1.47 |  |
|  |  | Upper Bound | 2.39 |  |
|  | 5% Trimmed Mean | | 1.87 |  |
|  | Median | | 1.00 |  |
|  | Variance | | 1.402 |  |
|  | Std. Deviation | | 1.184 |  |
|  | Minimum | | 1 |  |
|  | Maximum | | 4 |  |
|  | Range | | 3 |  |
|  | Interquartile Range | | 2 |  |
|  | Skewness | | .868 | .441 |
|  | Kurtosis | | -.843 | .858 |
| Hard_Finding_Position_Soft Lump | Mean | | 1.39 | .094 |
|  | 95% Confidence Interval for Mean | Lower Bound | 1.20 |  |
|  |  | Upper Bound | 1.59 |  |
|  | 5% Trimmed Mean | | 1.38 |  |
|  | Median | | 1.00 |  |
|  | Variance | | .247 |  |
|  | Std. Deviation | | .497 |  |
|  | Minimum | | 1 |  |
|  | Maximum | | 2 |  |
|  | Range | | 1 |  |
|  | Interquartile Range | | 1 |  |
|  | Skewness | | .464 | .441 |
|  | Kurtosis | | -1.928 | .858 |

### Confidence in Finding Position and Shape of the Irregularity

Participants reported their degree of agreement to a statement using a Likert Scale from 1 (Totally disagree) to 5 (Totally agree). The statement was: I am confident that I found the position and shape of the irregularity.

| *Descriptives* | | | | |
| --- | --- | --- | --- | --- |
|  | | | Statistic | Std. Error |
| Annulus | Mean | | 4.50 | .189 |
|  | 95% Confidence Interval for Mean | Lower Bound | 4.11 |  |
|  |  | Upper Bound | 4.89 |  |
|  | 5% Trimmed Mean | | 4.65 |  |
|  | Median | | 5.00 |  |
|  | Variance | | 1.000 |  |
|  | Std. Deviation | | 1.000 |  |
|  | Minimum | | 1 |  |
|  | Maximum | | 5 |  |
|  | Range | | 4 |  |
|  | Interquartile Range | | 1 |  |
|  | Skewness | | -2.393 | .441 |
|  | Kurtosis | | 5.692 | .858 |
| Hard Lump | Mean | | 3.93 | .235 |
|  | 95% Confidence Interval for Mean | Lower Bound | 3.45 |  |
|  |  | Upper Bound | 4.41 |  |
|  | 5% Trimmed Mean | | 3.98 |  |
|  | Median | | 4.50 |  |
|  | Variance | | 1.550 |  |
|  | Std. Deviation | | 1.245 |  |
|  | Minimum | | 2 |  |
|  | Maximum | | 5 |  |
|  | Range | | 3 |  |
|  | Interquartile Range | | 2 |  |
|  | Skewness | | -.600 | .441 |
|  | Kurtosis | | -1.354 | .858 |
| Soft Lump | Mean | | 4.36 | .187 |
|  | 95% Confidence Interval for Mean | Lower Bound | 3.97 |  |
|  |  | Upper Bound | 4.74 |  |
|  | 5% Trimmed Mean | | 4.49 |  |
|  | Median | | 5.00 |  |
|  | Variance | | .979 |  |
|  | Std. Deviation | | .989 |  |
|  | Minimum | | 1 |  |
|  | Maximum | | 5 |  |
|  | Range | | 4 |  |
|  | Interquartile Range | | 1 |  |
|  | Skewness | | -2.043 | .441 |
|  | Kurtosis | | 4.563 | .858 |

### Homogeneous Hardness

Participants reported their degree of agreement to a statement using a Likert Scale from 1 (Totally disagree) to 5 (Totally agree). The statement was: The irregularity had a constant/homogeneous hardness.

| *Descriptives* | | | | |
| --- | --- | --- | --- | --- |
|  | | | Statistic | Std. Error |
| Annulus | Mean | | 3.75 | .270 |
|  | 95% Confidence Interval for Mean | Lower Bound | 3.20 |  |
|  |  | Upper Bound | 4.30 |  |
|  | 5% Trimmed Mean | | 3.83 |  |
|  | Median | | 4.00 |  |
|  | Variance | | 2.046 |  |
|  | Std. Deviation | | 1.430 |  |
|  | Minimum | | 1 |  |
|  | Maximum | | 5 |  |
|  | Range | | 4 |  |
|  | Interquartile Range | | 3 |  |
|  | Skewness | | -.669 | .441 |
|  | Kurtosis | | -1.121 | .858 |
| Hard Lump | Mean | | 3.39 | .238 |
|  | 95% Confidence Interval for Mean | Lower Bound | 2.91 |  |
|  |  | Upper Bound | 3.88 |  |
|  | 5% Trimmed Mean | | 3.42 |  |
|  | Median | | 3.50 |  |
|  | Variance | | 1.581 |  |
|  | Std. Deviation | | 1.257 |  |
|  | Minimum | | 1 |  |
|  | Maximum | | 5 |  |
|  | Range | | 4 |  |
|  | Interquartile Range | | 3 |  |
|  | Skewness | | -.100 | .441 |
|  | Kurtosis | | -1.315 | .858 |
| Soft Lump | Mean | | 3.18 | .236 |
|  | 95% Confidence Interval for Mean | Lower Bound | 2.69 |  |
|  |  | Upper Bound | 3.66 |  |
|  | 5% Trimmed Mean | | 3.18 |  |
|  | Median | | 3.00 |  |
|  | Variance | | 1.560 |  |
|  | Std. Deviation | | 1.249 |  |
|  | Minimum | | 1 |  |
|  | Maximum | | 5 |  |
|  | Range | | 4 |  |
|  | Interquartile Range | | 2 |  |
|  | Skewness | | .129 | .441 |
|  | Kurtosis | | -1.416 | .858 |

### Self-assessed improvement in palpation

To get a measure of the potential learning effect occurring during the experiments, after completing the experiment, participants evaluated the statement: I became better at finding the irregularity during the experiment. A Likert Scale from 1 (Totally disagree) to 5 (Totally agree) was used.

Table 7 Palpation Improvement

| *Descriptives Palpation Improvement* | | | | | |
| --- | --- | --- | --- | --- | --- |
|  | | Frequency | Percent | Valid Percent | Cumulative Percent |
| Valid | 1 | 1 | 3.6 | 3.6 | 3.6 |
|  | 2 | 2 | 7.1 | 7.1 | 10.7 |
|  | 3 | 5 | 17.9 | 17.9 | 28.6 |
|  | 4 | 10 | 35.7 | 35.7 | 64.3 |
|  | 5 | 10 | 35.7 | 35.7 | 100.0 |
|  | Total | 28 | 100.0 | 100.0 |  |

# Area and diameters of the palpable irregularities

From the binarized images we can extract area measurements for each lump condition and for each of the 28 samples. In table 8. Means, standard deviation and standard error is calculated for these measurements. Further, by assuming circular geometries for the lump conditions and circular and a mean radius equal the radius of the magnet configuration for the annulus condition, we can approximate diameters for the three geometries. Also mean, standard deviation and standard error is calculated for these approximated diameters.

Table 8 Descriptive statistics of area and approximated diameters for the lump geometries

| **Hard Lump** | | **Soft Lump** | | **Annulus** | | |
| --- | --- | --- | --- | --- | --- | --- |
| **Mean Area [mm^2]** | **Lump Mean Dia[mm]** | **Mean Area [mm^2]** | **Mean Dia** | **Mean Area [mm^2]** | **Mean DiaOut [mm]** | **Mean DiaIn [mm]** |
| 7911.92 | 100.32 | 8094.62 | 100.32 | 4915.08 | 82.97 | 29.50 |
| **SD Area [mm^2]** | **SD Dia[mm]** | **SD Area [mm^2]** | **SD Dia[mm]** | **SD Area [mm^2]** | **SD DiaOut[mm]** | **SD DiaIn[mm]** |
| 473.98 | 3.06 | 641.40 | 3.06 | 370.56 | 2.18 | 4.61 |
| **SE Area [mm^2]** | **SE Dia[mm]** | **SE Area [mm^2]** | **SE Dia[mm]** | **SE Area [mm^2]** | **SE DiaOut[mm]** | **SE DiaIn[mm]** |
| 89.57 | 0.57 | 121.21 | 0.58 | 70.03 | 0.41 | 0.87 |

# Instructions given to participants

Thank you for participating in this testing. The prototype is a part of my master degree within product development at TrollLabs and NTNU. You will now do a test on the prototype. You are going to search for a possible irregularity within a field. There is a possibility that there are no irregularity. The test consists of four scenarios where the position, form and hardness of the irregularity can change. It is the irregularity you are going to describe the hardness of, and not the material above or below. You will get the same questions after each scenario.

*Before each scenario:

The scenario is ready. You are going to search with your hands after an irregularity and when you are certain of the location and form of the irregularity, let me know. Start when you are feeling ready. [Starting timer when participant touches the field.] [Stop timer when participants signals that they are finished.]

[If they say that they found an irregularity, place a plastic sheet over the palpation field.] Draw the contour of the irregularity and place an x within the area(s) of the irregularity. Now you shall estimate the hardness. To do that, compare the hardness of the irregularity with the hardness of the objects here. [After hardness comparison, the participant fills out a questionnaire.]

[If they did not find any irregularity, arrange the next scenario after the participant have moved away from the prototype.]

Additional info: the participants were told to use their hands to palpate. Regarding palpation technique, nothing was specified. Most of the participants used both their hands.
